# Supplementary figures and images for: A conserved mycobacterial nucleomodulin hijacks the host COMPASS complex to reprogram pro-inflammatory transcription and promote intracellular survival (part 1 of 2)
Source: eLife. 2026 Mar 31;14:RP107677. doi: 10.7554/eLife.107677 (PMC13038263; doi:10.7554/eLife.107677)

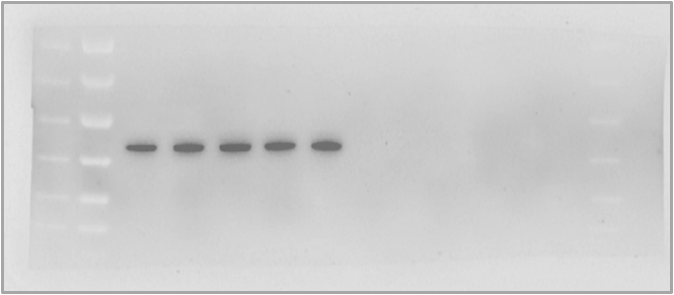

Supplement: Figure 2—source data 1. [file elife-107677-fig2-data1.zip › Figure 2E-b-actin-1.tif]

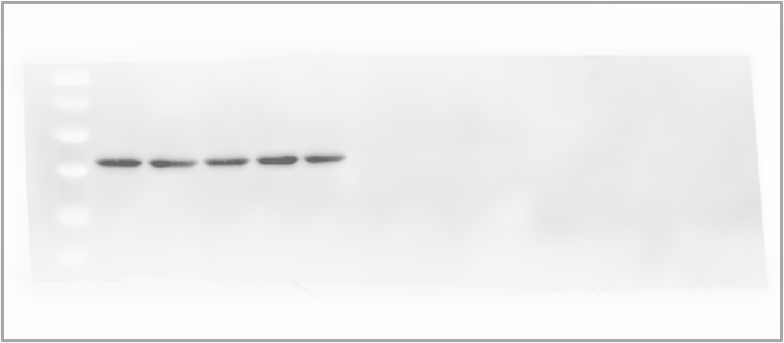

Supplement: Figure 2—source data 1. [file elife-107677-fig2-data1.zip › Figure 2E-b-actin-2.tif]

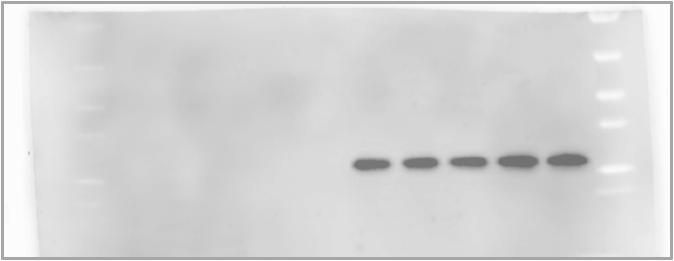

Supplement: Figure 2—source data 1. [file elife-107677-fig2-data1.zip › Figure 2E-Histone H3-1.tif]

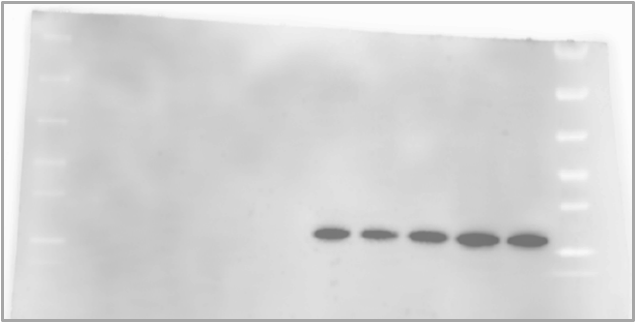

Supplement: Figure 2—source data 1. [file elife-107677-fig2-data1.zip › Figure 2E-Histone H3-2.tif]

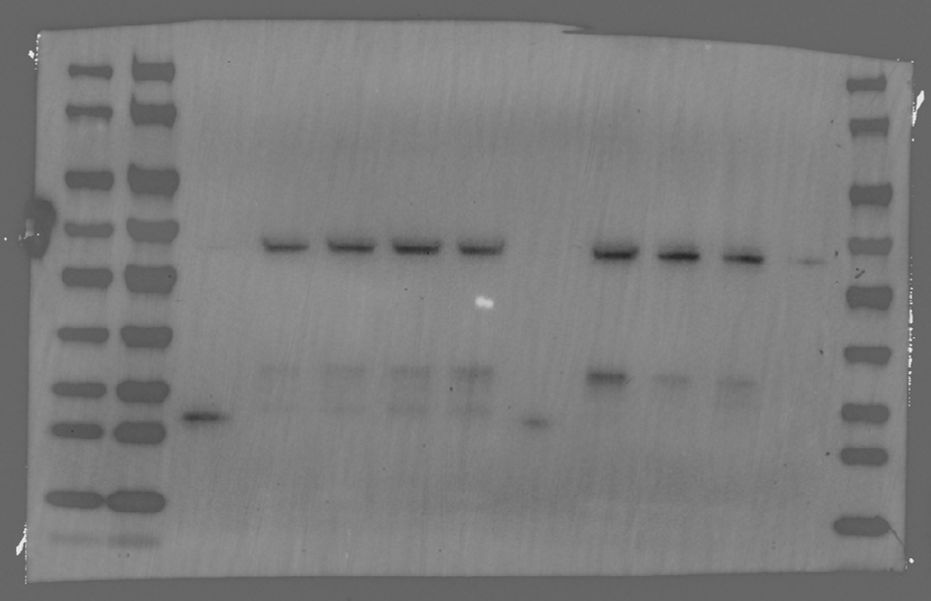

Supplement: Figure 2—source data 1. [file elife-107677-fig2-data1.zip › Figure 2E-MgdE-GFP-1.tif]

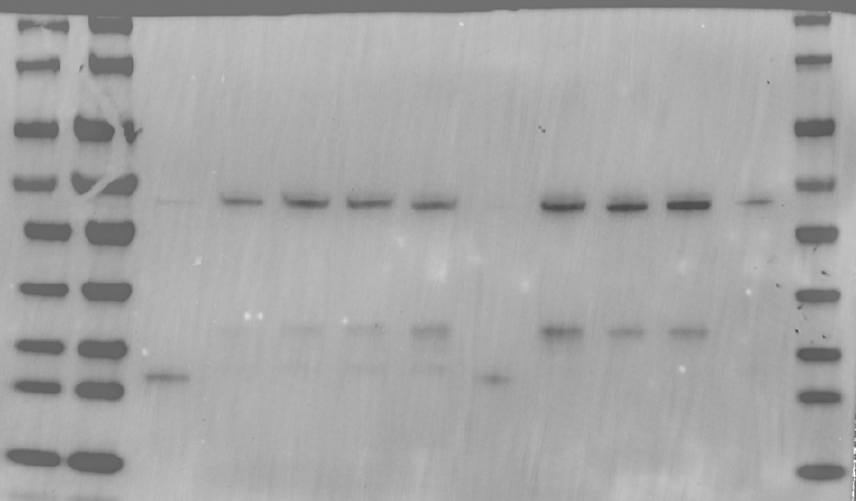

Supplement: Figure 2—source data 1. [file elife-107677-fig2-data1.zip › Figure 2E-MgdE-GFP-2.tif]

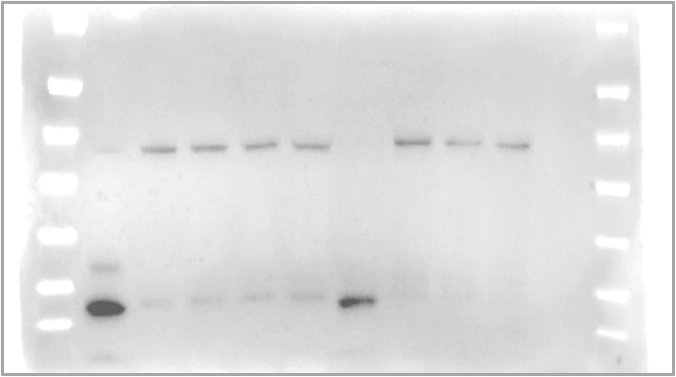

Supplement: Figure 2—source data 1. [file elife-107677-fig2-data1.zip › Figure 2E-MgdE-GFP-3.tif]

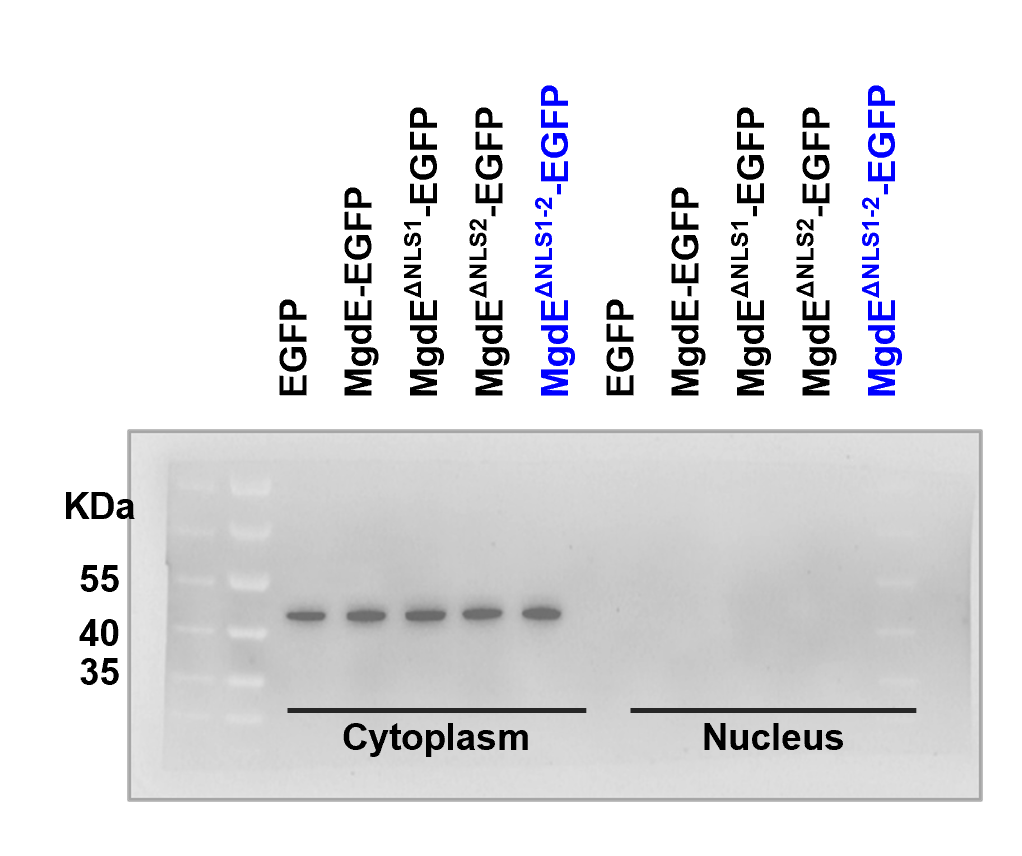

Supplement: Figure 2—source data 2. [file elife-107677-fig2-data2.zip › Figure 2E-b-actin.tif]

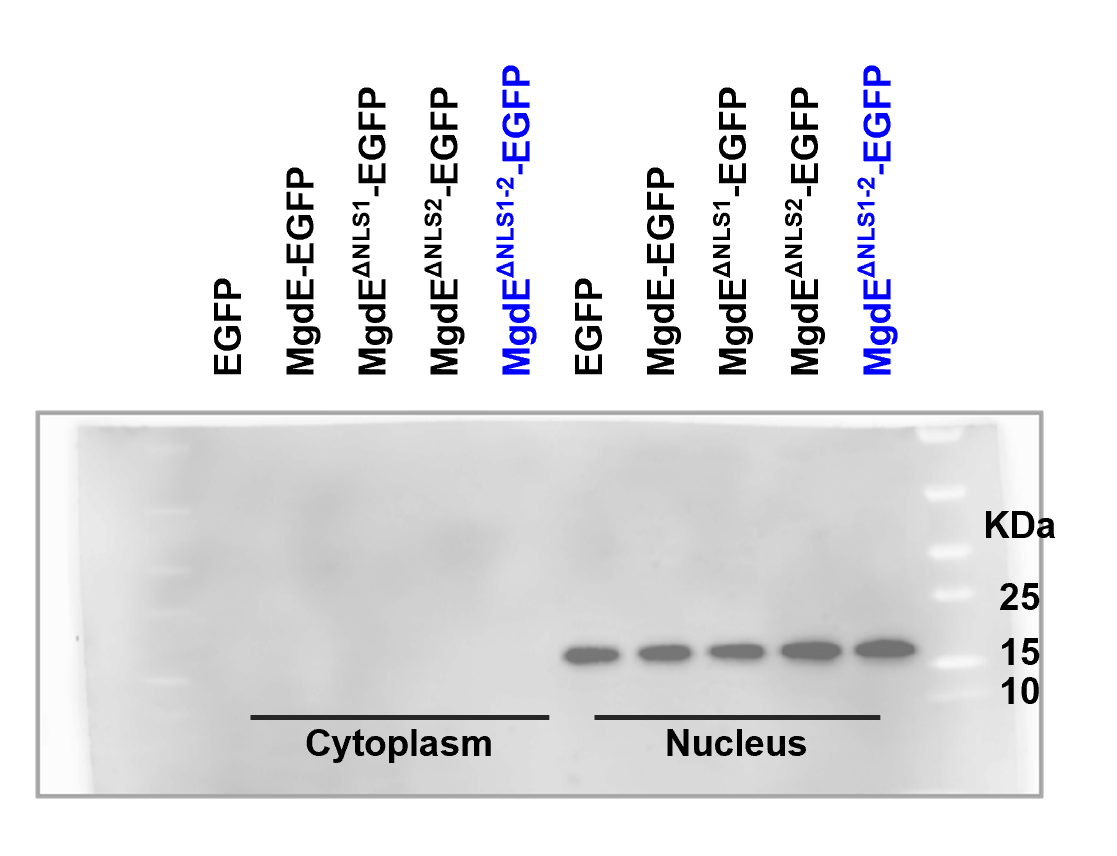

Supplement: Figure 2—source data 2. [file elife-107677-fig2-data2.zip › Figure 2E-Histone H3.tif]

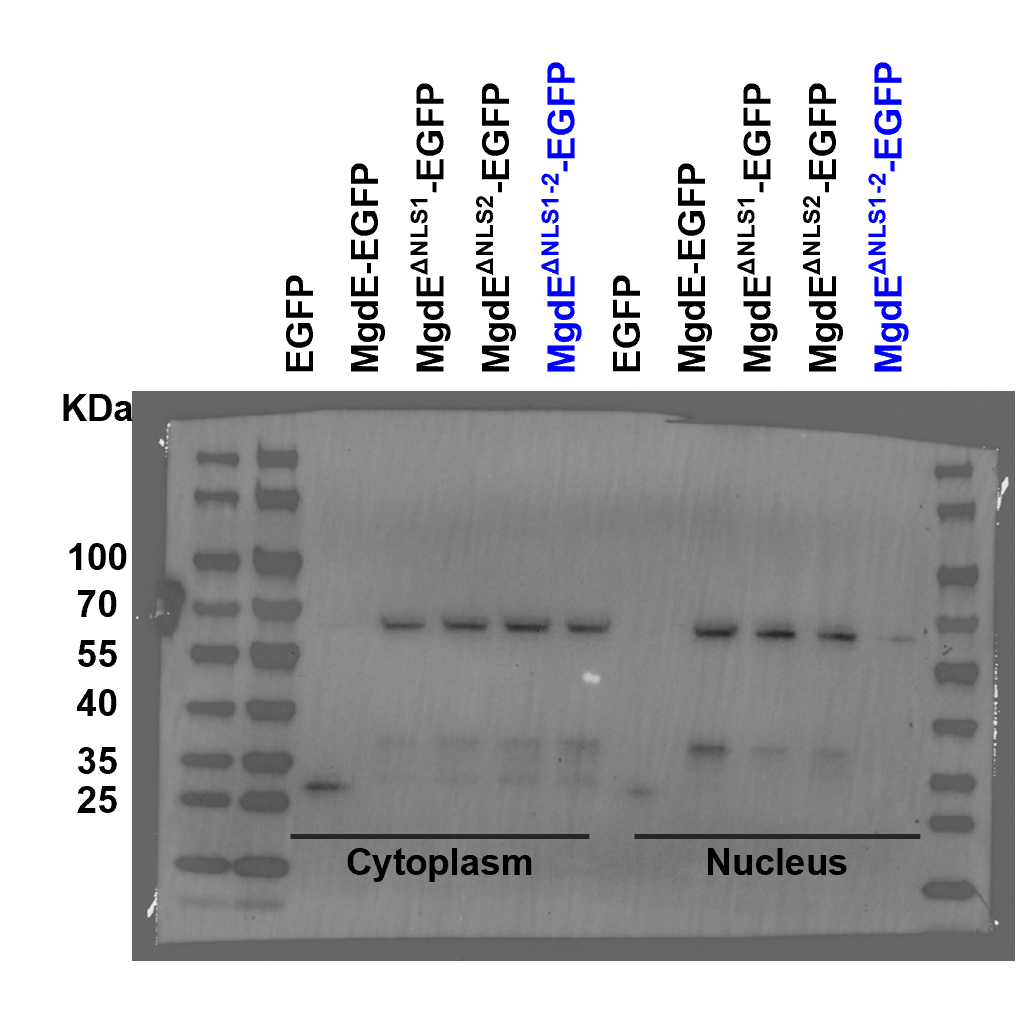

Supplement: Figure 2—source data 2. [file elife-107677-fig2-data2.zip › Figure 2E-MgdE-GFP-1.tif]

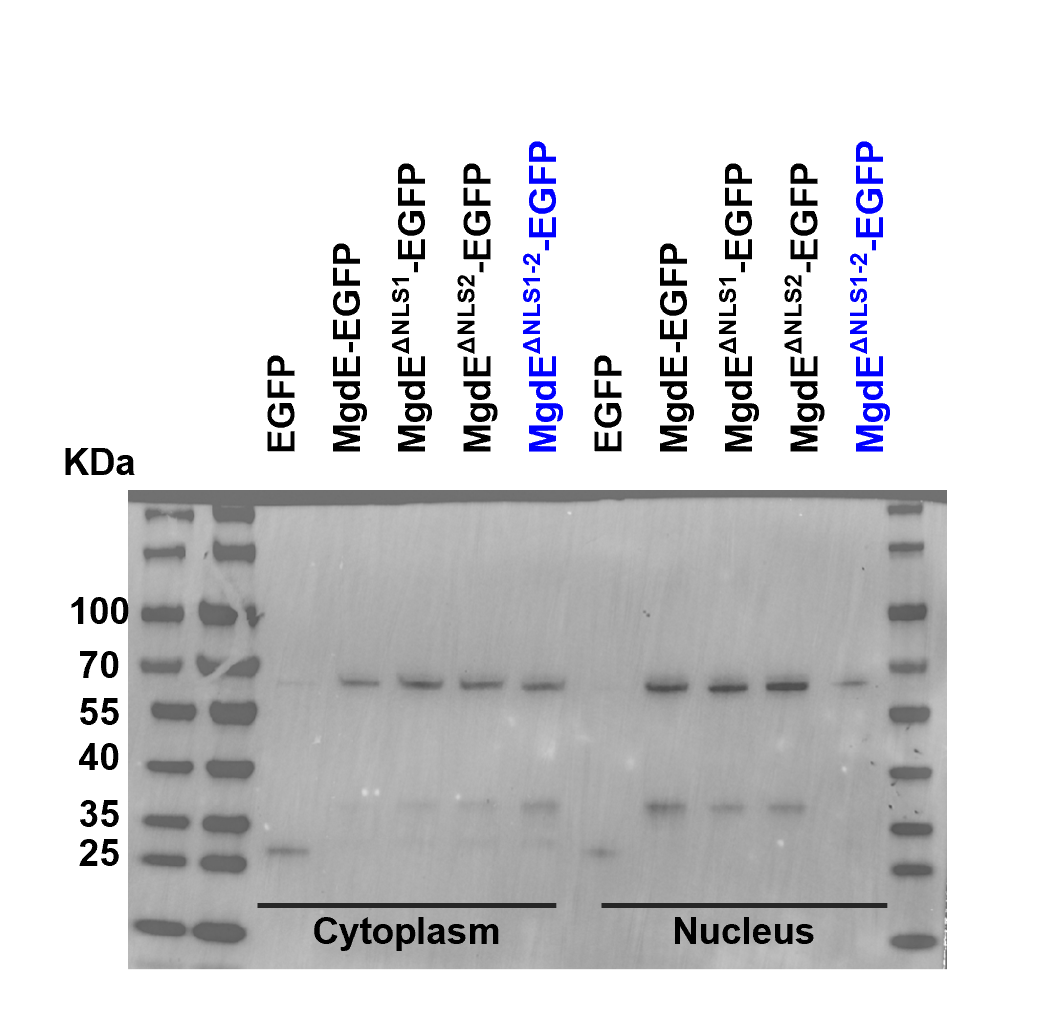

Supplement: Figure 2—source data 2. [file elife-107677-fig2-data2.zip › Figure 2E-MgdE-GFP-2.tif]

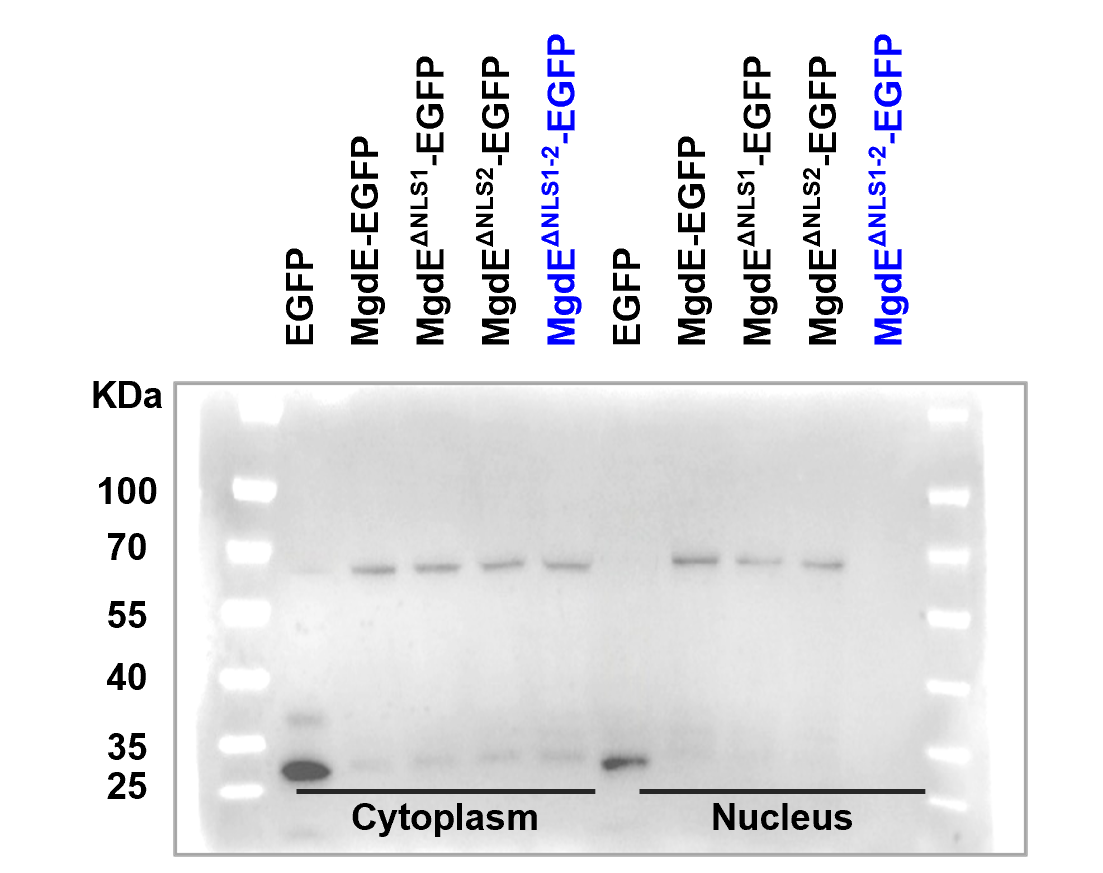

Supplement: Figure 2—source data 2. [file elife-107677-fig2-data2.zip › Figure 2E-MgdE-GFP-3.tif]

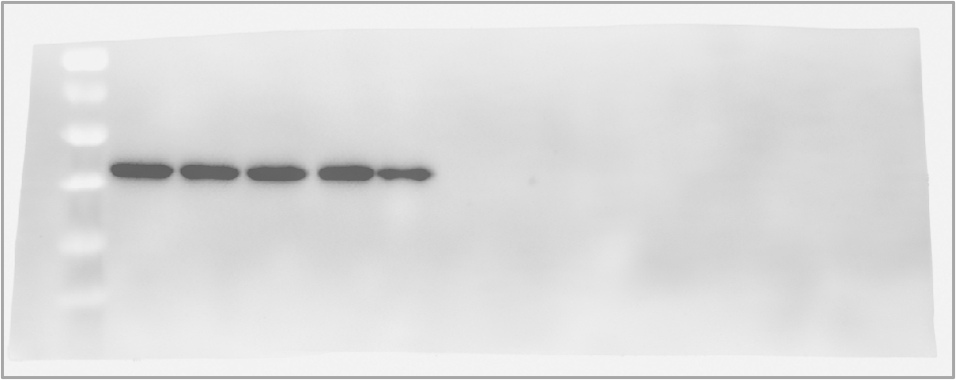

Supplement: Figure 2—source data 3. [file elife-107677-fig2-data3.zip › Figure 2—source data 3/Figure 2F-b-actin-1.tif]

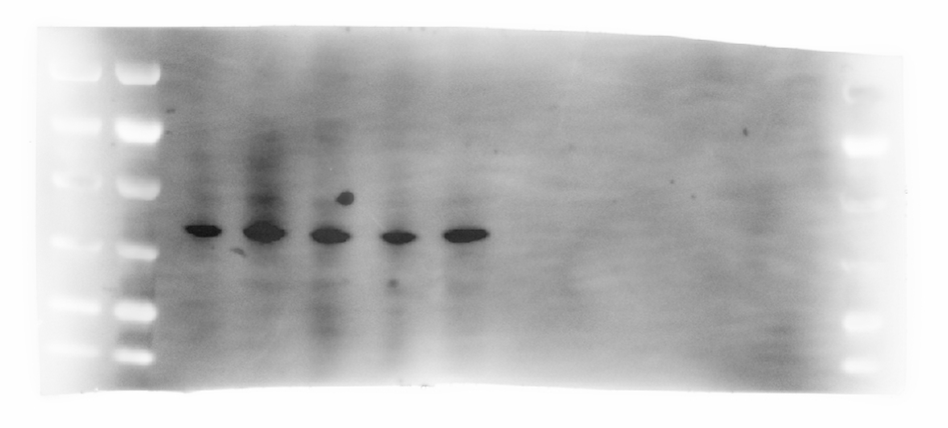

Supplement: Figure 2—source data 3. [file elife-107677-fig2-data3.zip › Figure 2—source data 3/Figure 2F-b-actin-2.tif]

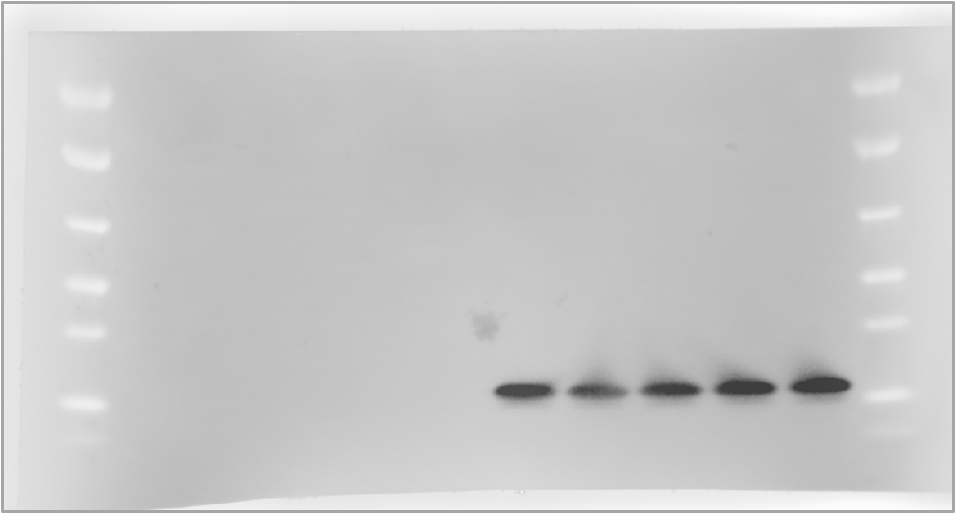

Supplement: Figure 2—source data 3. [file elife-107677-fig2-data3.zip › Figure 2—source data 3/Figure 2F-Histone H3-1.tif]

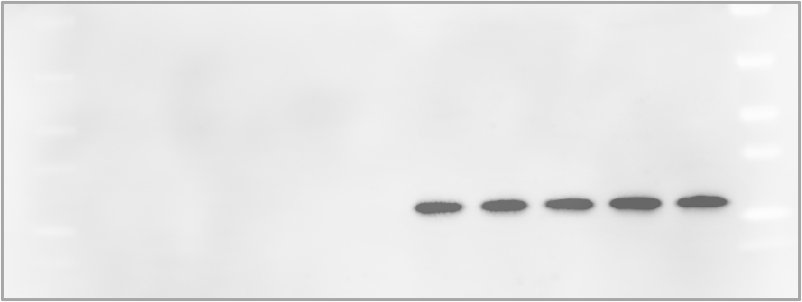

Supplement: Figure 2—source data 3. [file elife-107677-fig2-data3.zip › Figure 2—source data 3/Figure 2F-Histone H3-2.tif]

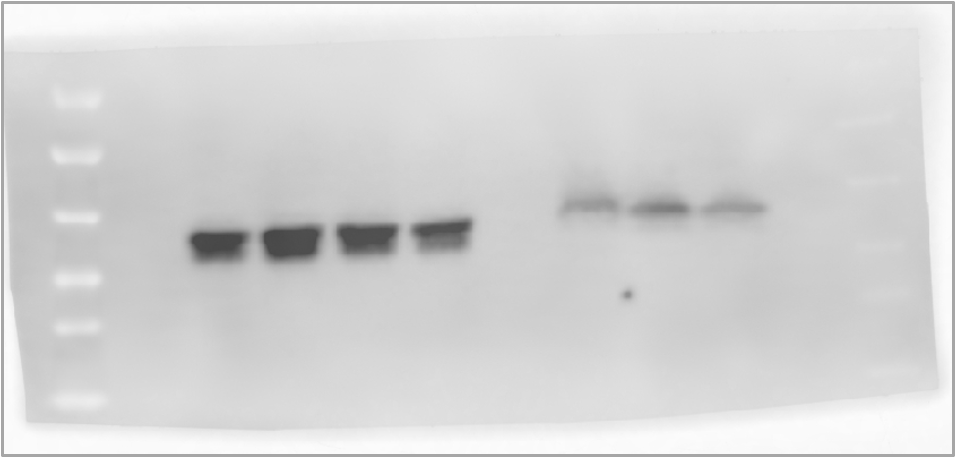

Supplement: Figure 2—source data 3. [file elife-107677-fig2-data3.zip › Figure 2—source data 3/Figure 2F-MgdE-Flag-1.tif]

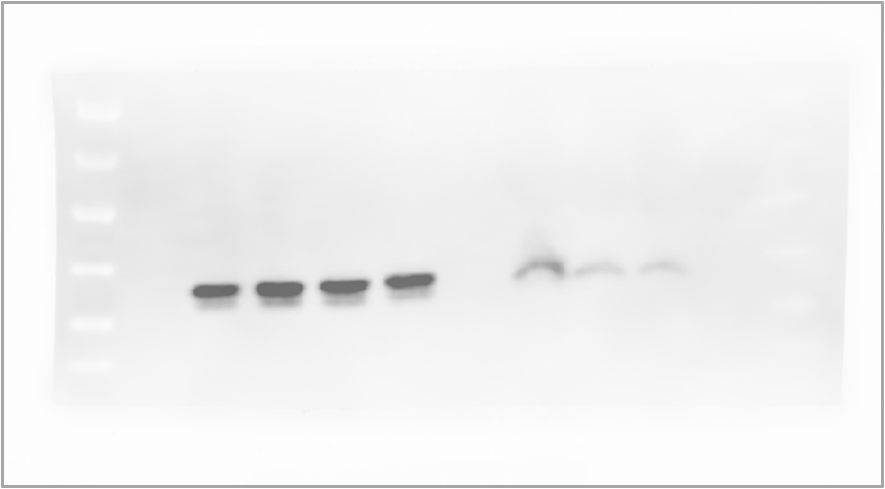

Supplement: Figure 2—source data 3. [file elife-107677-fig2-data3.zip › Figure 2—source data 3/Figure 2F-MgdE-Flag-2.tif]

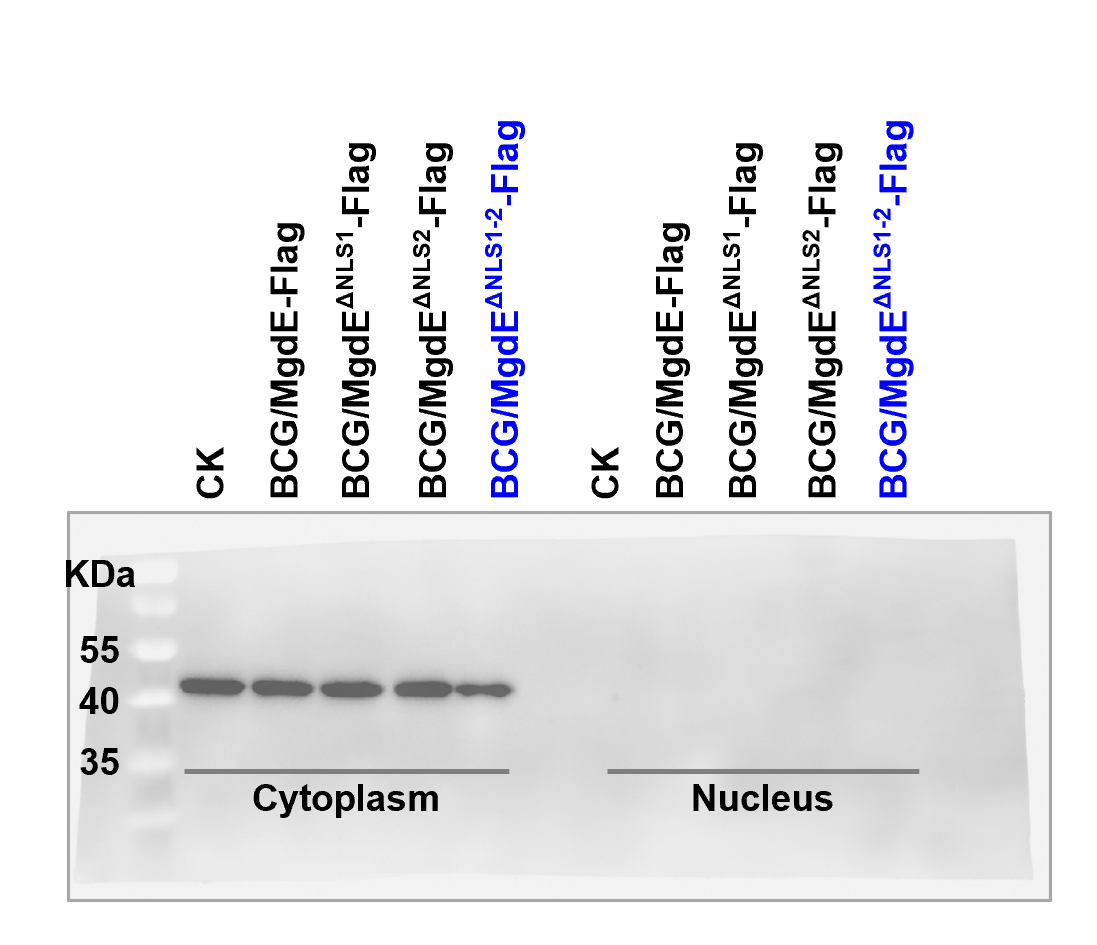

Supplement: Figure 2—source data 4. [file elife-107677-fig2-data4.zip › Figure 2—source data 4/Figure 2F-b-actin-1.tif]

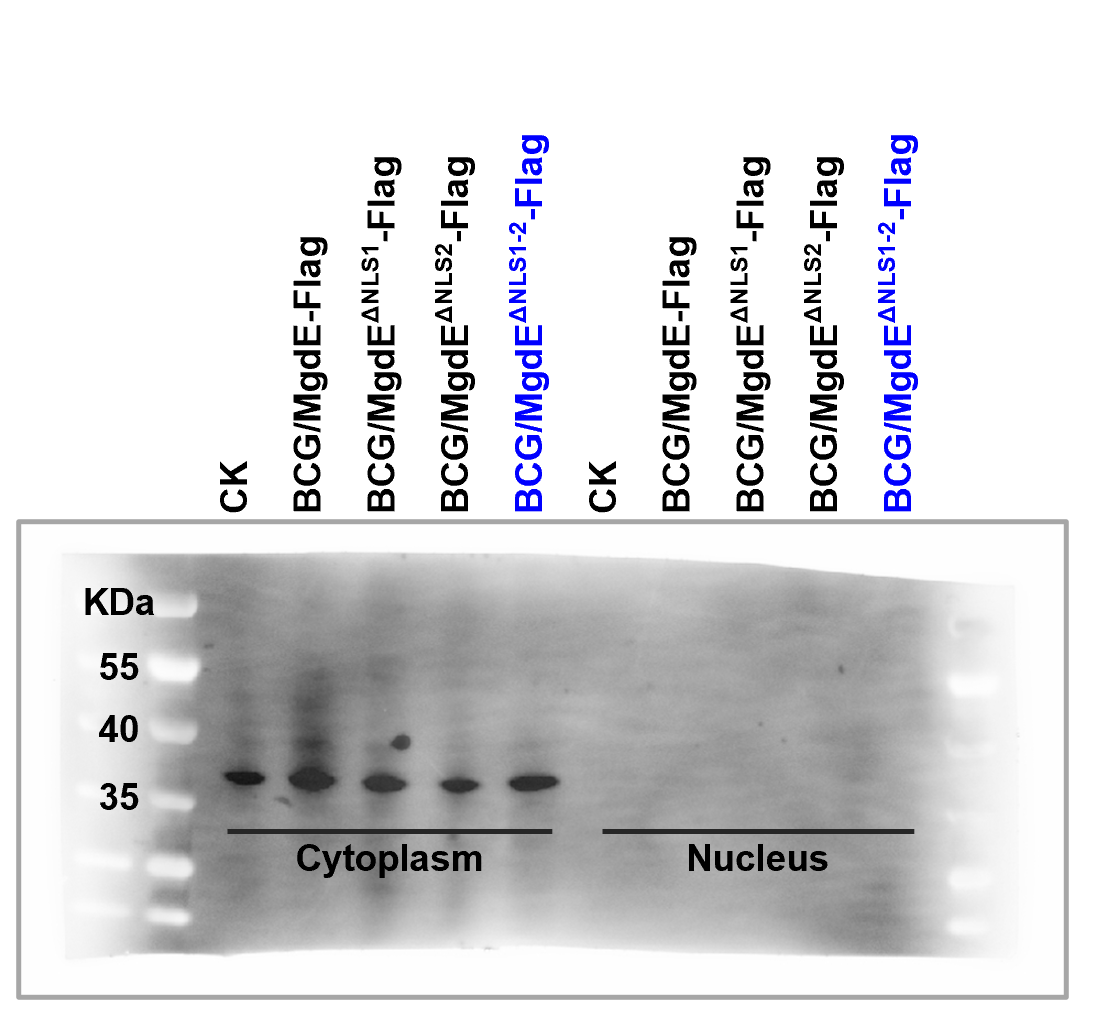

Supplement: Figure 2—source data 4. [file elife-107677-fig2-data4.zip › Figure 2—source data 4/Figure 2F-b-actin-2.tif]

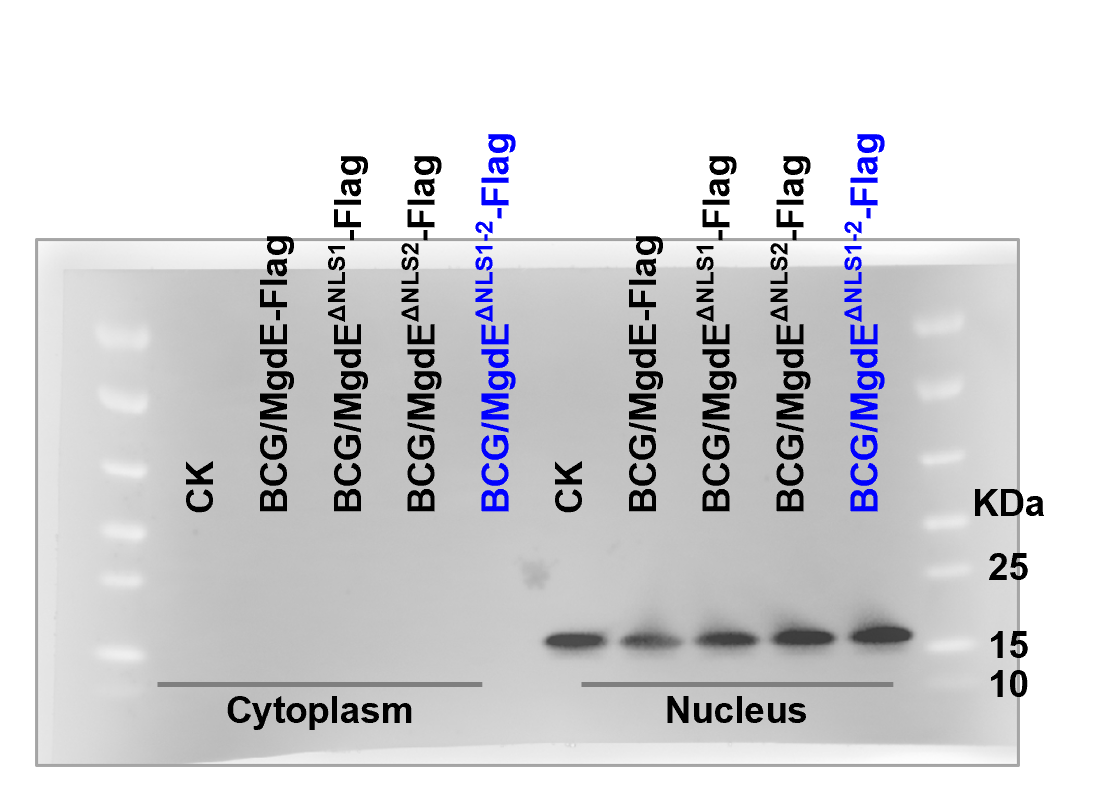

Supplement: Figure 2—source data 4. [file elife-107677-fig2-data4.zip › Figure 2—source data 4/Figure 2F-Histone H3-1.tif]

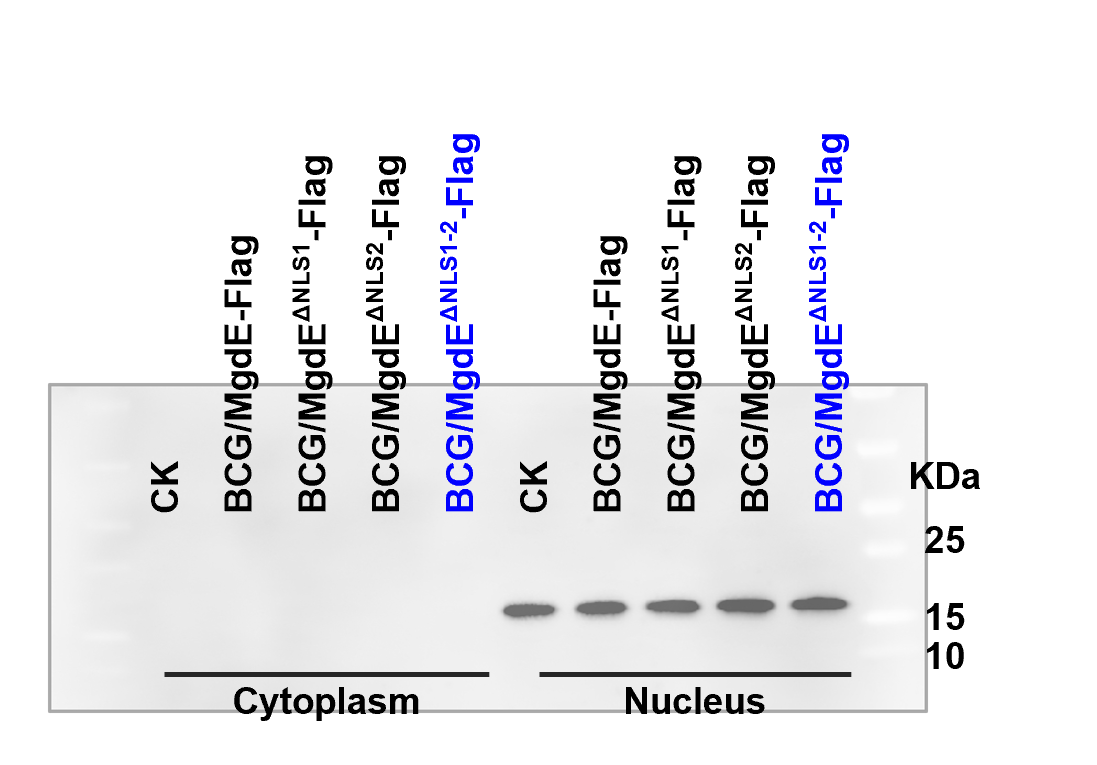

Supplement: Figure 2—source data 4. [file elife-107677-fig2-data4.zip › Figure 2—source data 4/Figure 2F-Histone H3-2.tif]

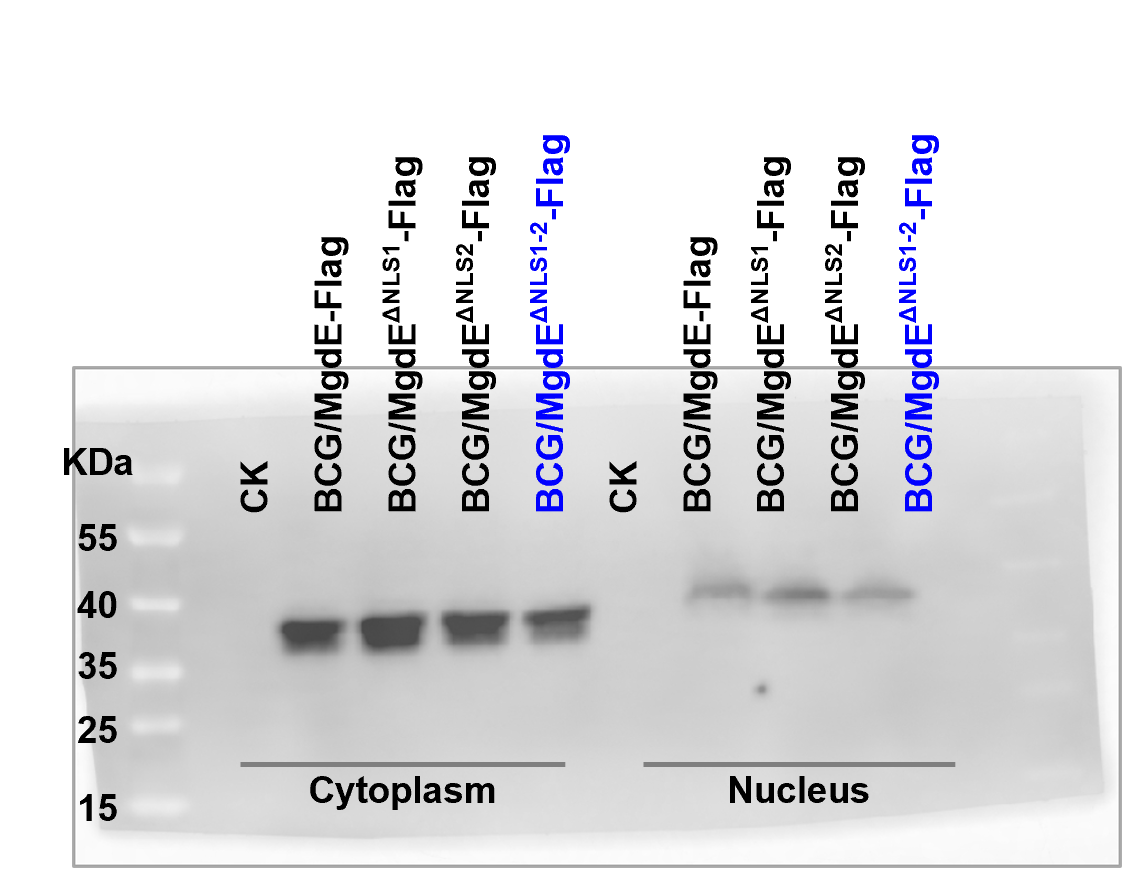

Supplement: Figure 2—source data 4. [file elife-107677-fig2-data4.zip › Figure 2—source data 4/Figure 2F-MgdE-Flag-1.tif]

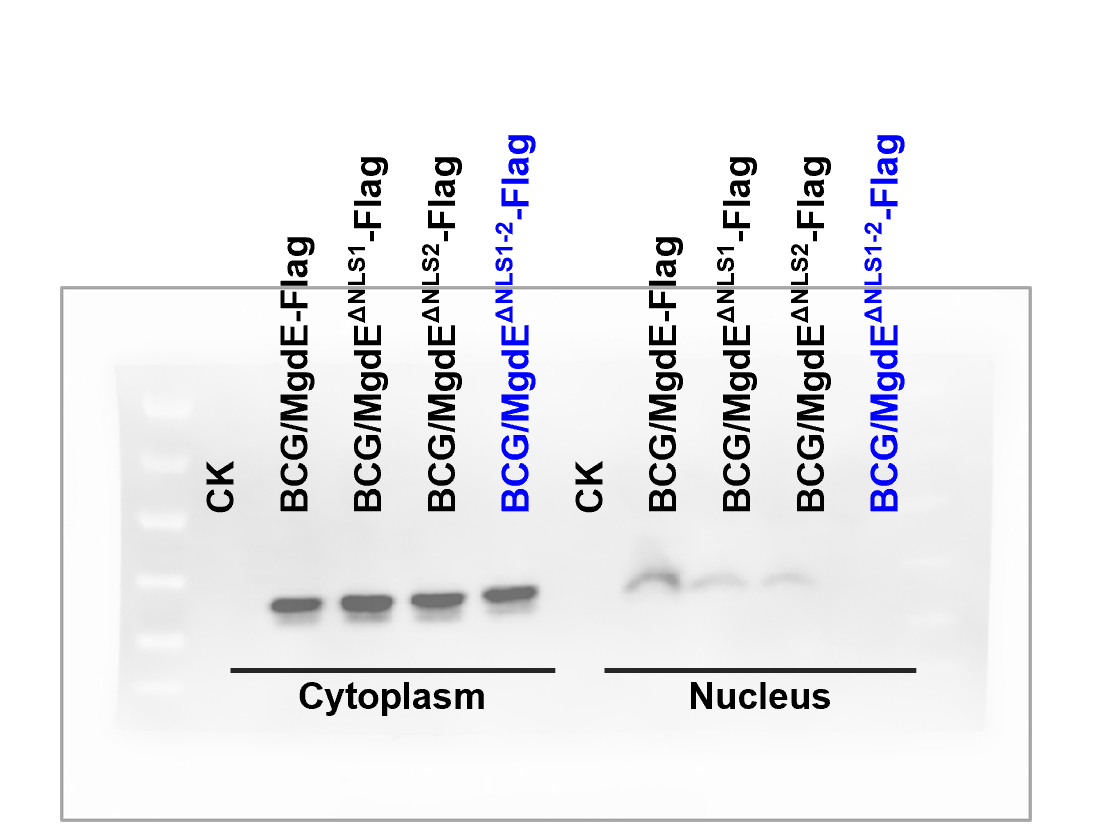

Supplement: Figure 2—source data 4. [file elife-107677-fig2-data4.zip › Figure 2—source data 4/Figure 2F-MgdE-Flag-2.tif]

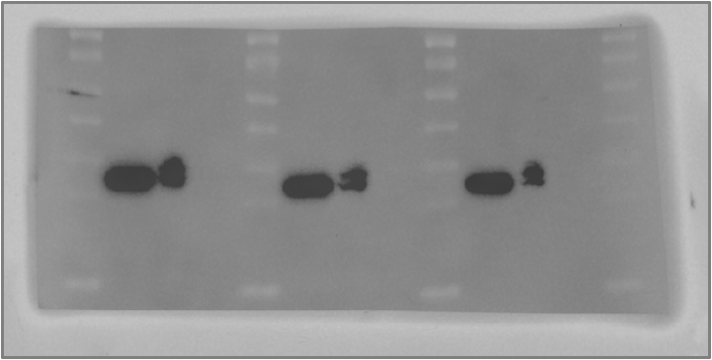

Supplement: Figure 2—figure supplement 1—source data 1. [file elife-107677-fig2-figsupp1-data1.zip › Figure2-figure supplement 2A-Ag85B.tif]

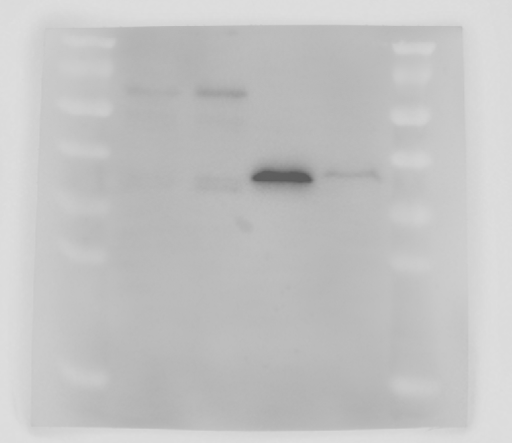

Supplement: Figure 2—figure supplement 1—source data 1. [file elife-107677-fig2-figsupp1-data1.zip › Figure2-figure supplement 2A-MgdE-Flag.tif]

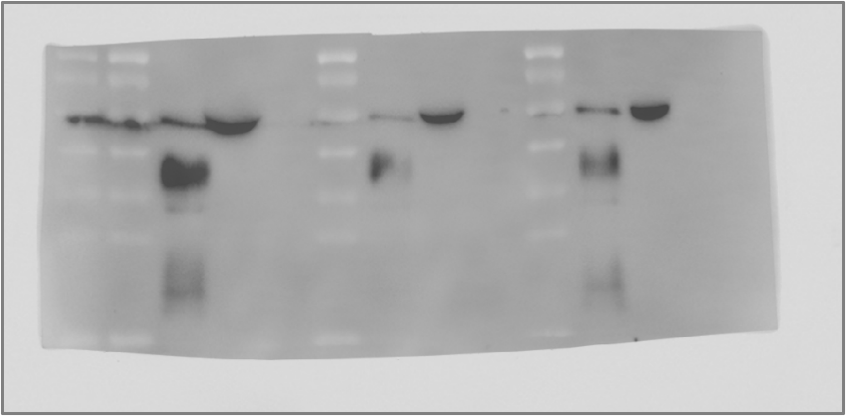

Supplement: Figure 2—figure supplement 1—source data 1. [file elife-107677-fig2-figsupp1-data1.zip › Figure2-figure supplement 2A-MgdE-GlpX.tif]

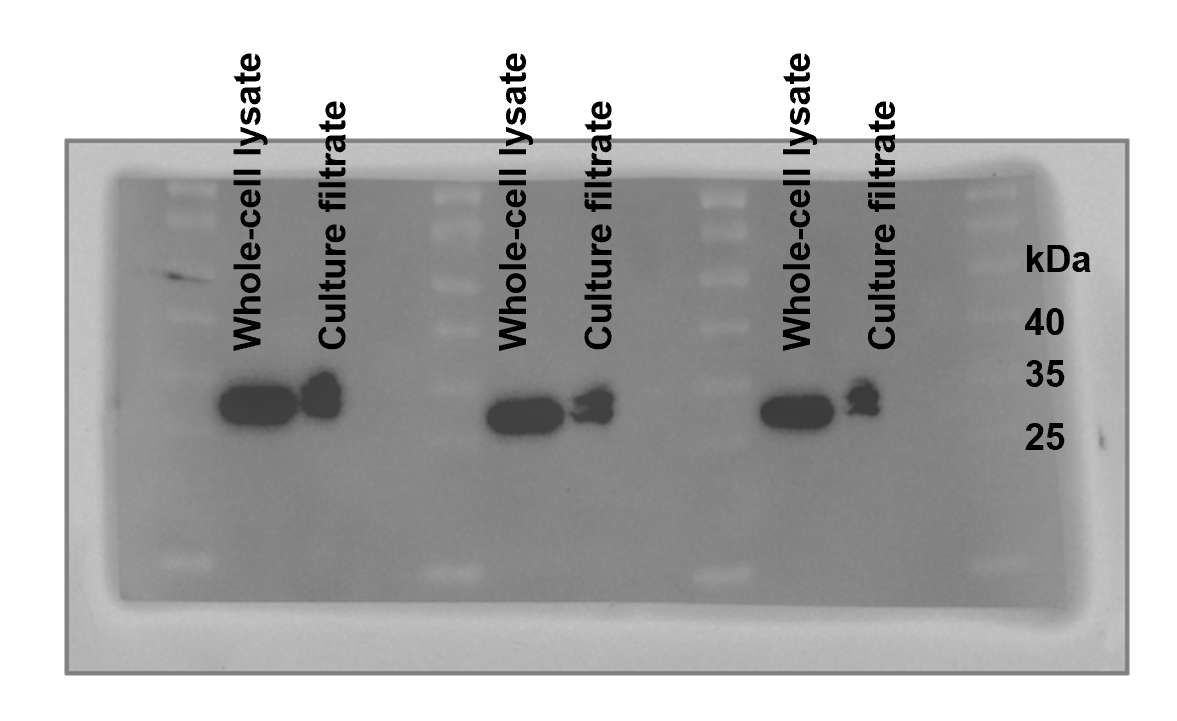

Supplement: Figure 2—figure supplement 1—source data 2. [file elife-107677-fig2-figsupp1-data2.zip › Figure2-figure supplement 2A-Ag85B.tif]

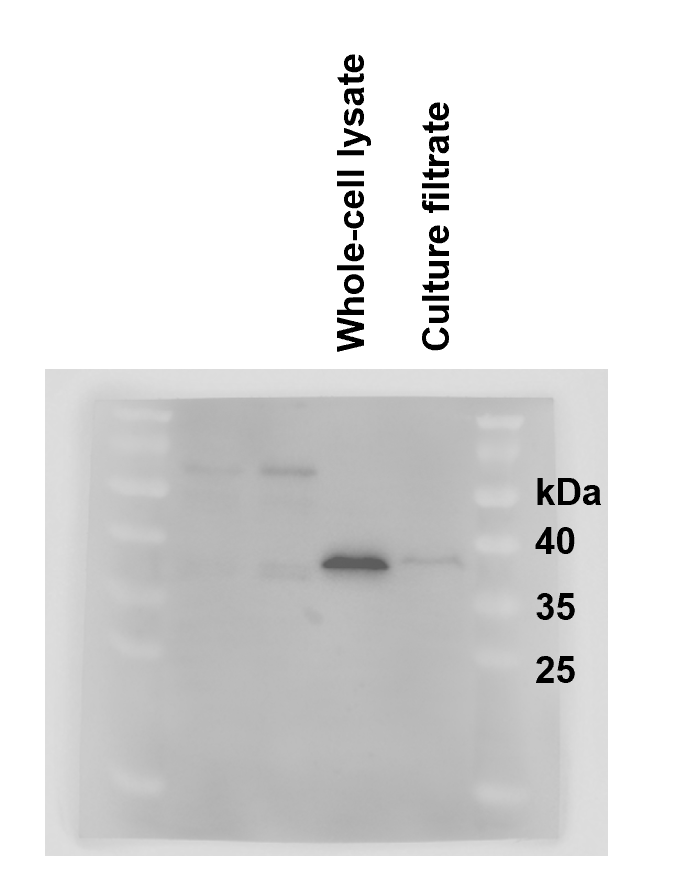

Supplement: Figure 2—figure supplement 1—source data 2. [file elife-107677-fig2-figsupp1-data2.zip › Figure2-figure supplement 2A-MgdE-Flag.tif]

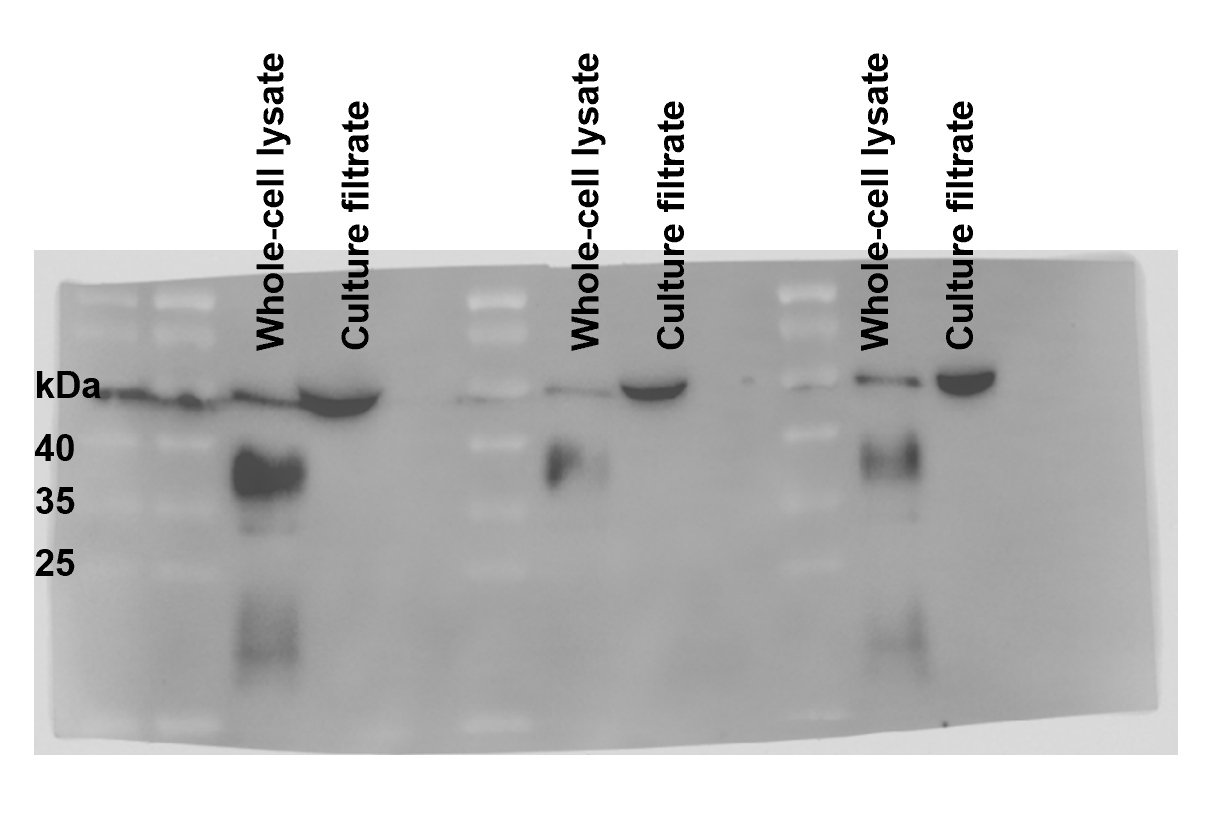

Supplement: Figure 2—figure supplement 1—source data 2. [file elife-107677-fig2-figsupp1-data2.zip › Figure2-figure supplement 2A-MgdE-GlpX.tif]

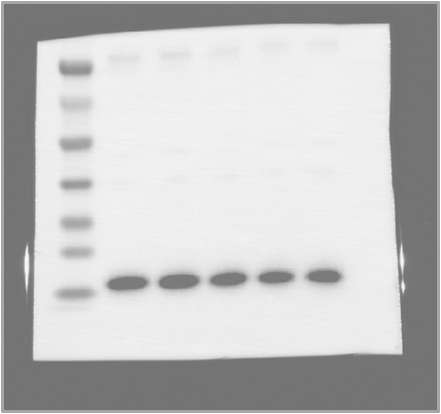

Supplement: Figure 2—figure supplement 1—source data 3. [file elife-107677-fig2-figsupp1-data3.zip › Figure2-figure supplement—source data 3/Figure2-figure supplement 2E-Histone H3-1.tif]

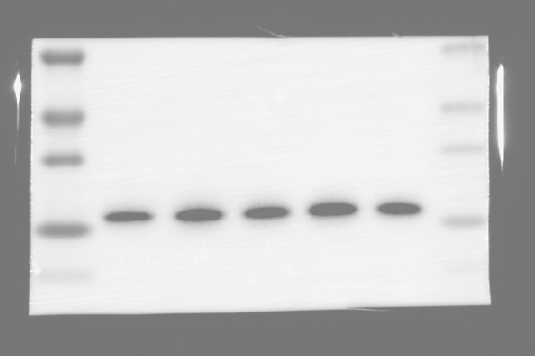

Supplement: Figure 2—figure supplement 1—source data 3. [file elife-107677-fig2-figsupp1-data3.zip › Figure2-figure supplement—source data 3/Figure2-figure supplement 2E-Histone H3-2.tif]

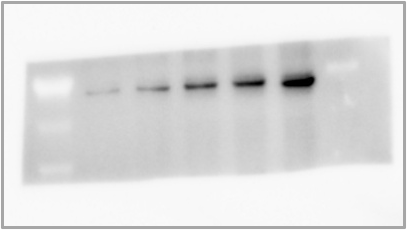

Supplement: Figure 2—figure supplement 1—source data 3. [file elife-107677-fig2-figsupp1-data3.zip › Figure2-figure supplement—source data 3/Figure2-figure supplement 2E-MgdE-EGFP-1.tif]

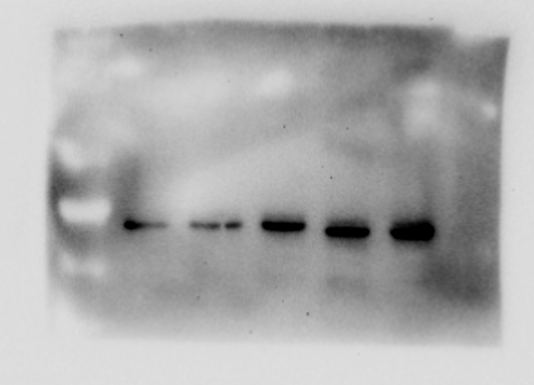

Supplement: Figure 2—figure supplement 1—source data 3. [file elife-107677-fig2-figsupp1-data3.zip › Figure2-figure supplement—source data 3/Figure2-figure supplement 2E-MgdE-EGFP-2.tif]

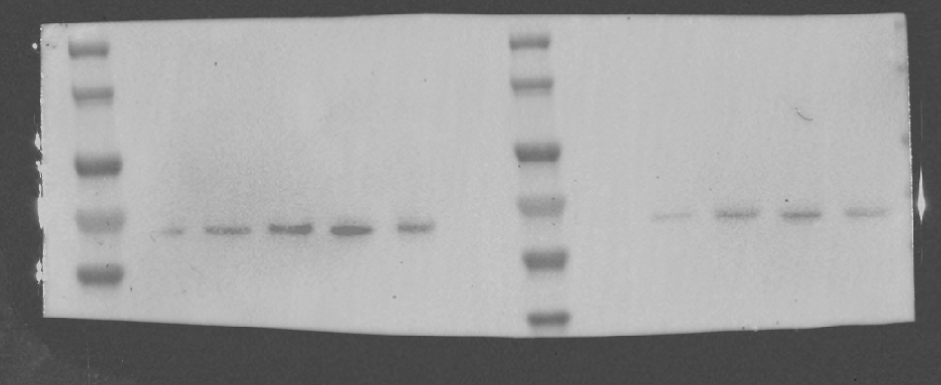

Supplement: Figure 2—figure supplement 1—source data 3. [file elife-107677-fig2-figsupp1-data3.zip › Figure2-figure supplement—source data 3/Figure2-figure supplement 2E-MgdE-EGFP-3.tif]

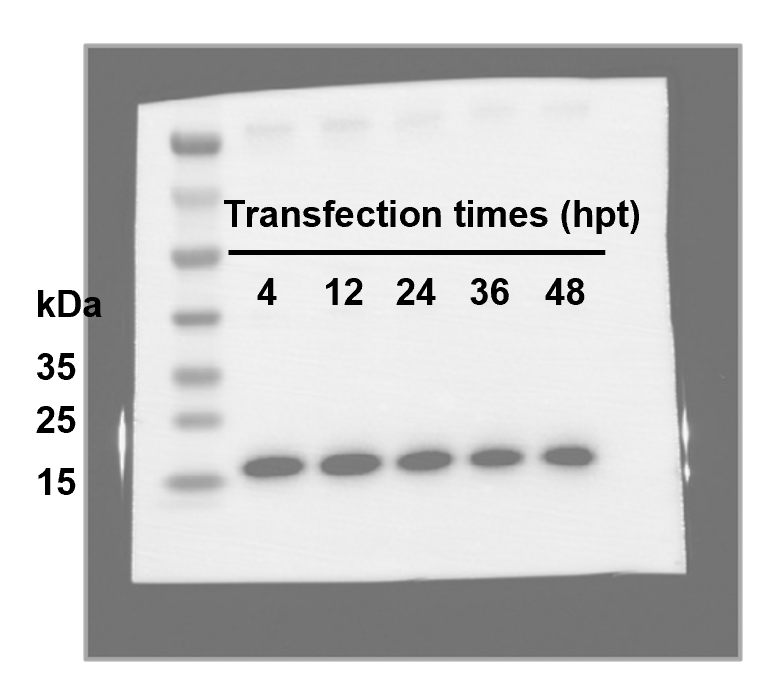

Supplement: Figure 2—figure supplement 1—source data 4. [file elife-107677-fig2-figsupp1-data4.zip › Figure2-figure supplement—source data 4/Figure2-figure supplement 2E-Histone H3-1.tif]

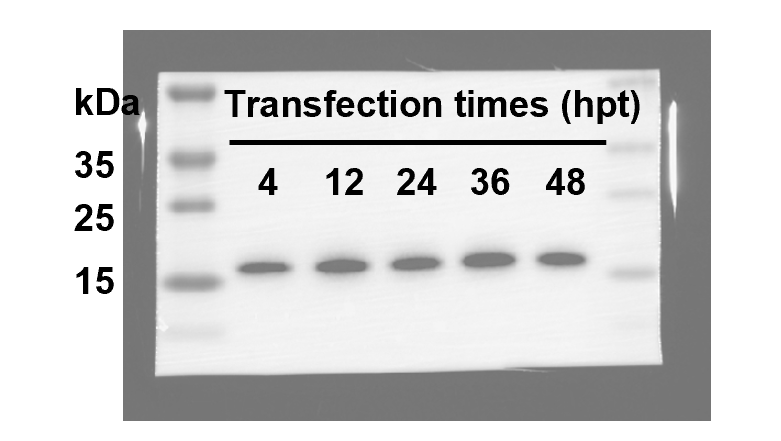

Supplement: Figure 2—figure supplement 1—source data 4. [file elife-107677-fig2-figsupp1-data4.zip › Figure2-figure supplement—source data 4/Figure2-figure supplement 2E-Histone H3-2.tif]

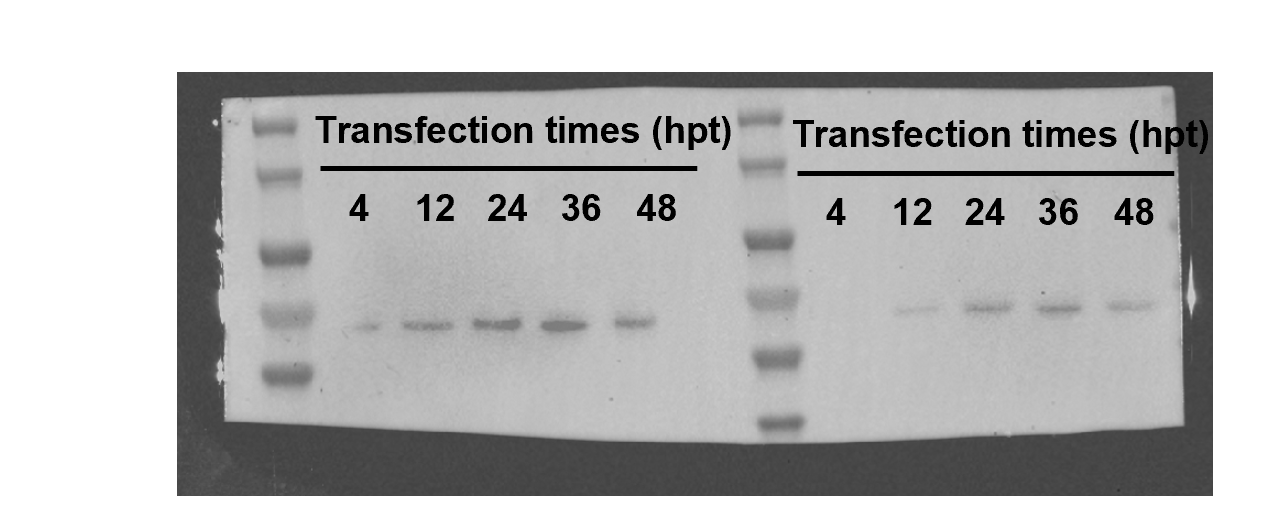

Supplement: Figure 2—figure supplement 1—source data 4. [file elife-107677-fig2-figsupp1-data4.zip › Figure2-figure supplement—source data 4/Figure2-figure supplement 2E-MgdE-EGFP-1.tif]

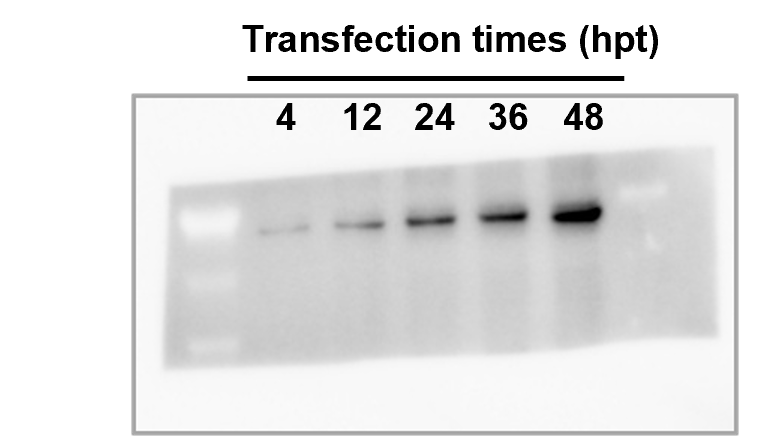

Supplement: Figure 2—figure supplement 1—source data 4. [file elife-107677-fig2-figsupp1-data4.zip › Figure2-figure supplement—source data 4/Figure2-figure supplement 2E-MgdE-EGFP-2.tif]

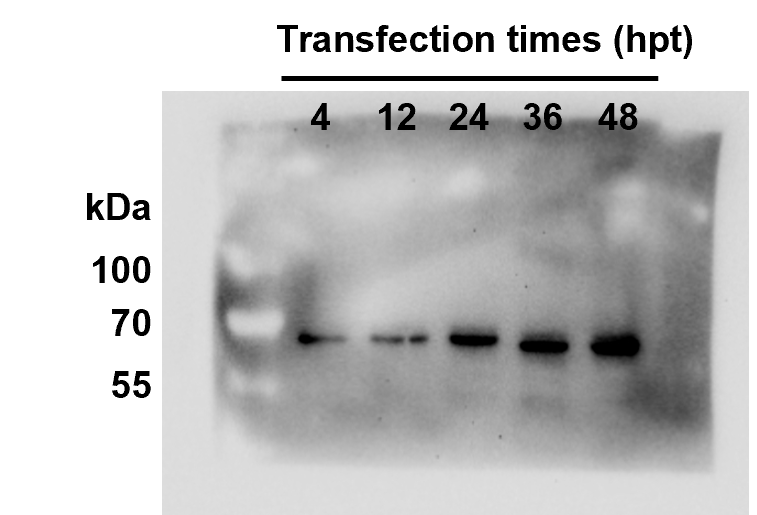

Supplement: Figure 2—figure supplement 1—source data 4. [file elife-107677-fig2-figsupp1-data4.zip › Figure2-figure supplement—source data 4/Figure2-figure supplement 2E-MgdE-EGFP-3.tif]

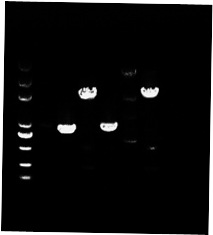

Supplement: Figure 3—figure supplement 1—source data 1. [file elife-107677-fig3-figsupp1-data1.zip › Figure3-figure supplement—source data 1/Figure3-figure supplement 3B-PCR.tif]

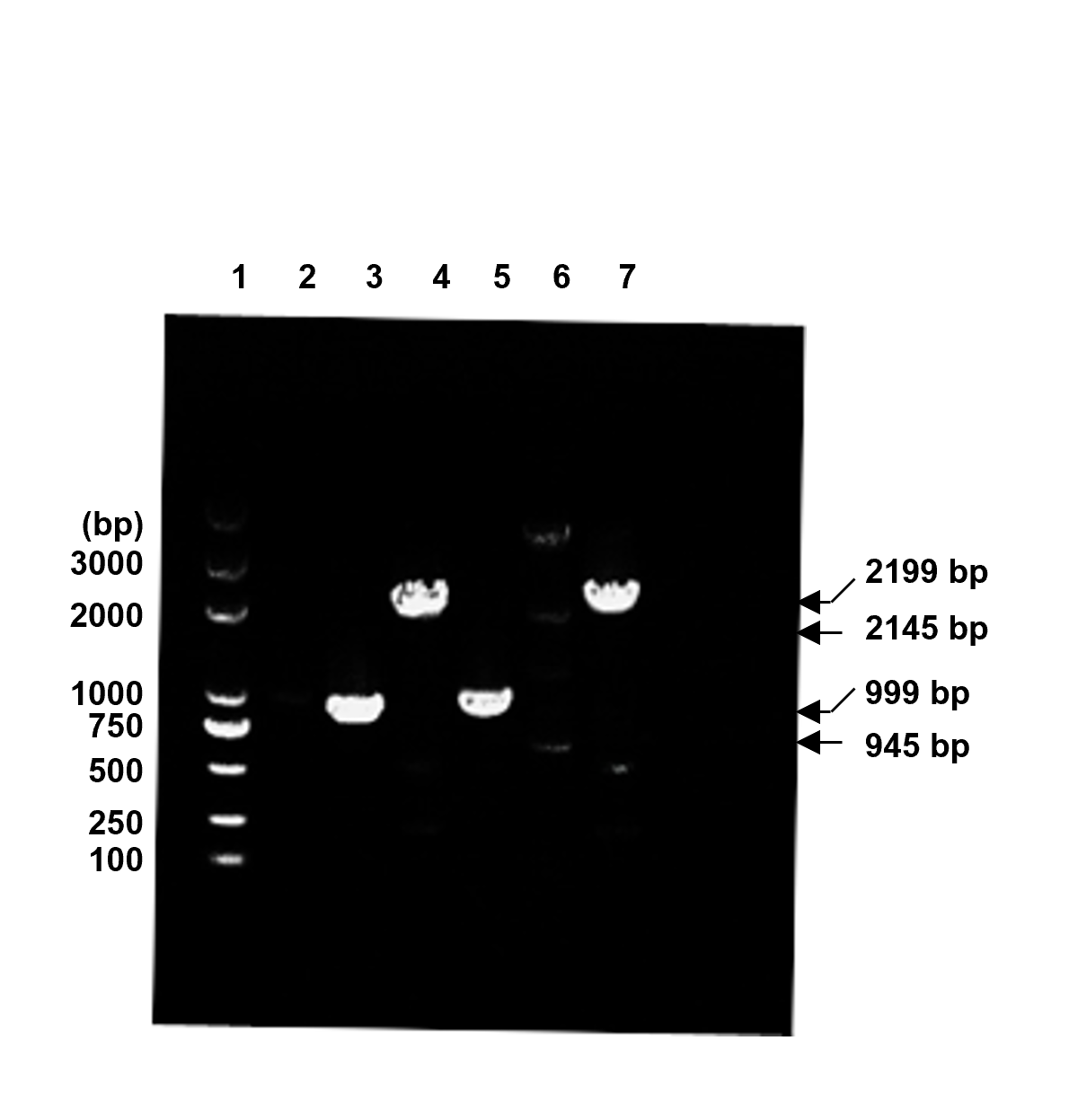

Supplement: Figure 3—figure supplement 1—source data 2. [file elife-107677-fig3-figsupp1-data2.zip › Figure3-figure supplement—source data 2/Figure3-figure supplement 3B-PCR.tif]

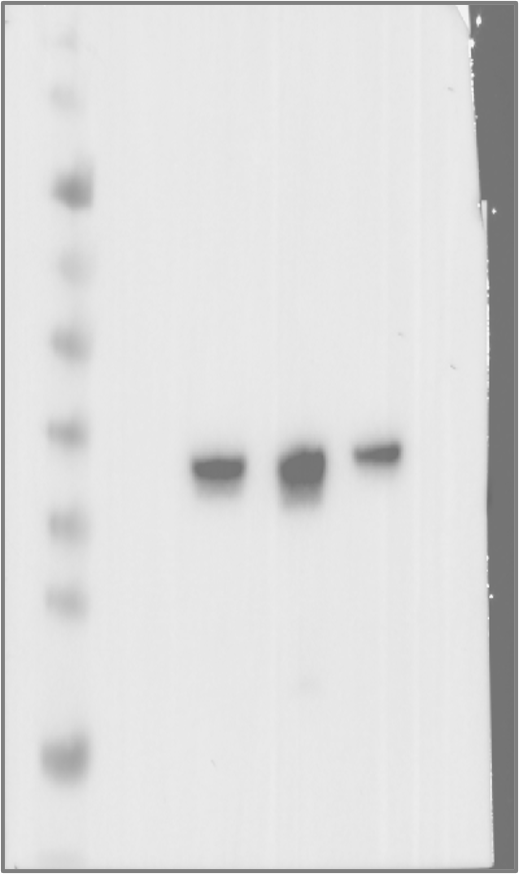

Supplement: Figure 4—source data 1. [file elife-107677-fig4-data1.zip › Figure 4B-Input-IB-Flag.tif]

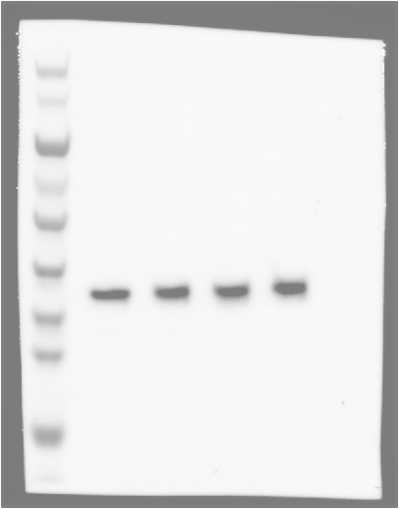

Supplement: Figure 4—source data 1. [file elife-107677-fig4-data1.zip › Figure 4B-Input-IB-GAPDH.tif]

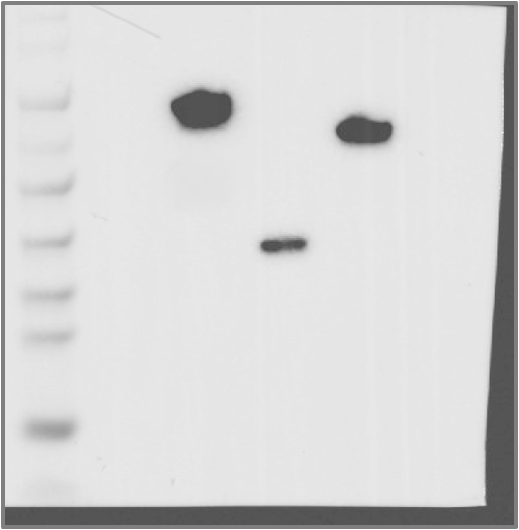

Supplement: Figure 4—source data 1. [file elife-107677-fig4-data1.zip › Figure 4B-Input-IB-HA.tif]

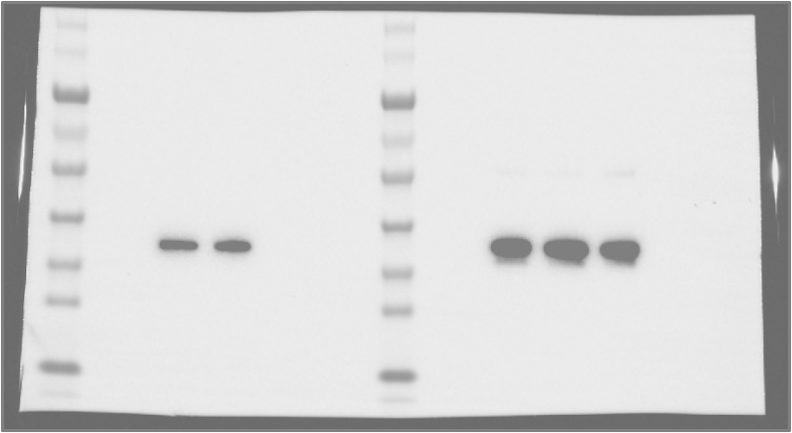

Supplement: Figure 4—source data 1. [file elife-107677-fig4-data1.zip › Figure 4B-IP-IB-Flag.tif]

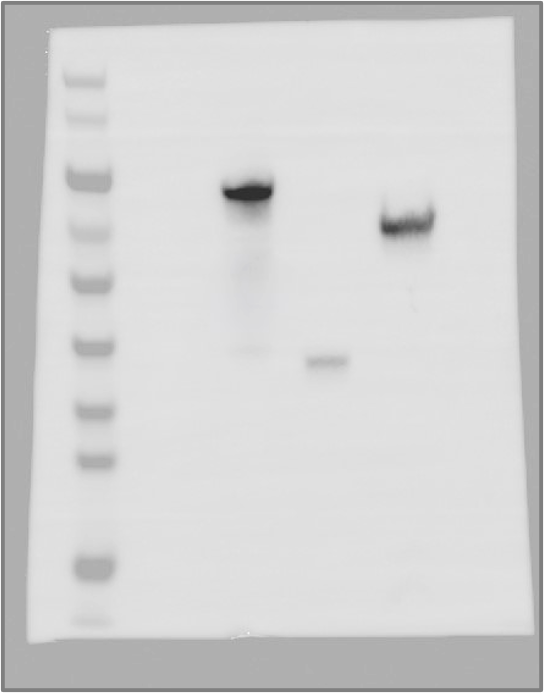

Supplement: Figure 4—source data 1. [file elife-107677-fig4-data1.zip › Figure 4B-IP-IB-HA.tif]

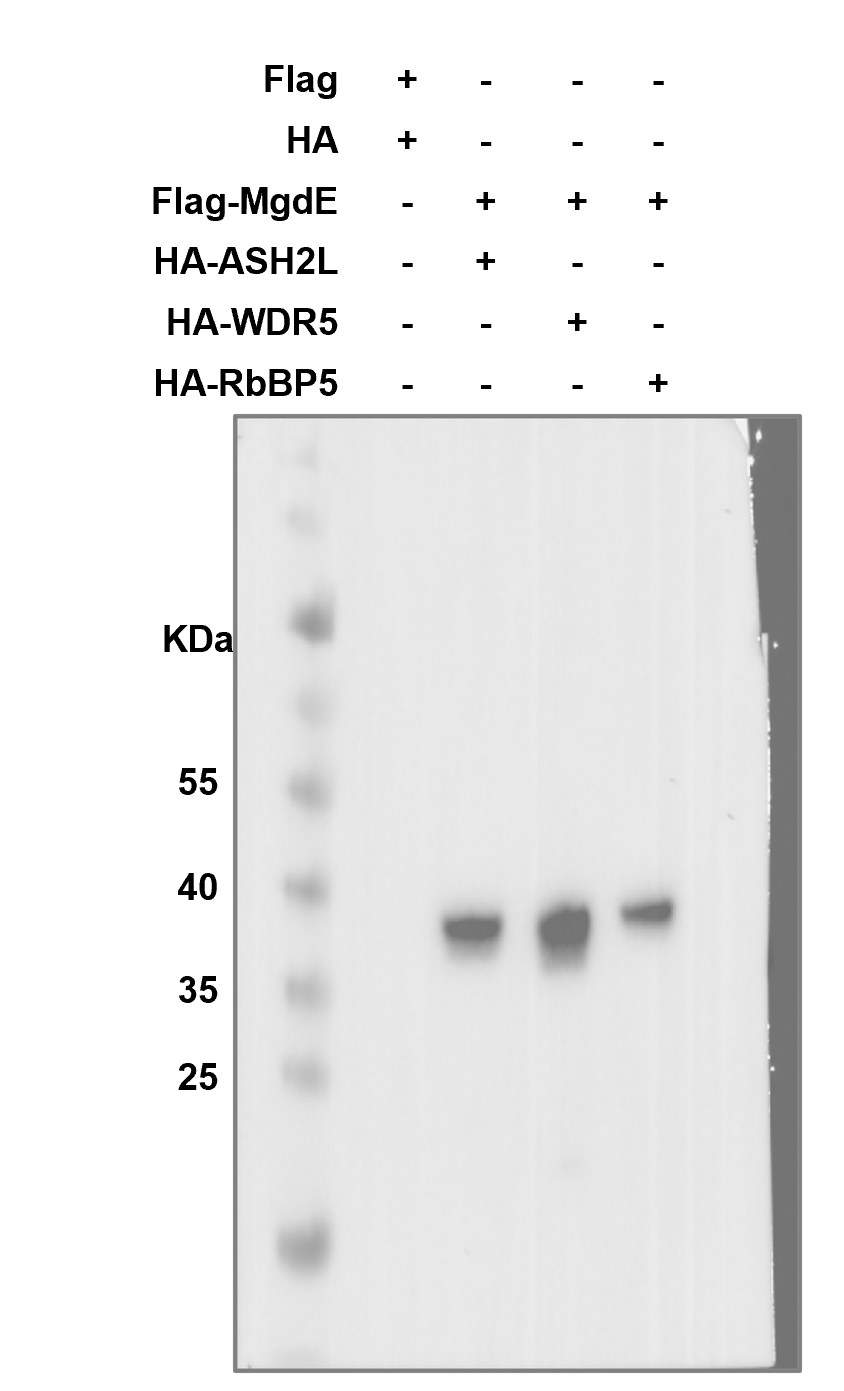

Supplement: Figure 4—source data 2. [file elife-107677-fig4-data2.zip › Figure 4B-Input-IB-Flag.tif]

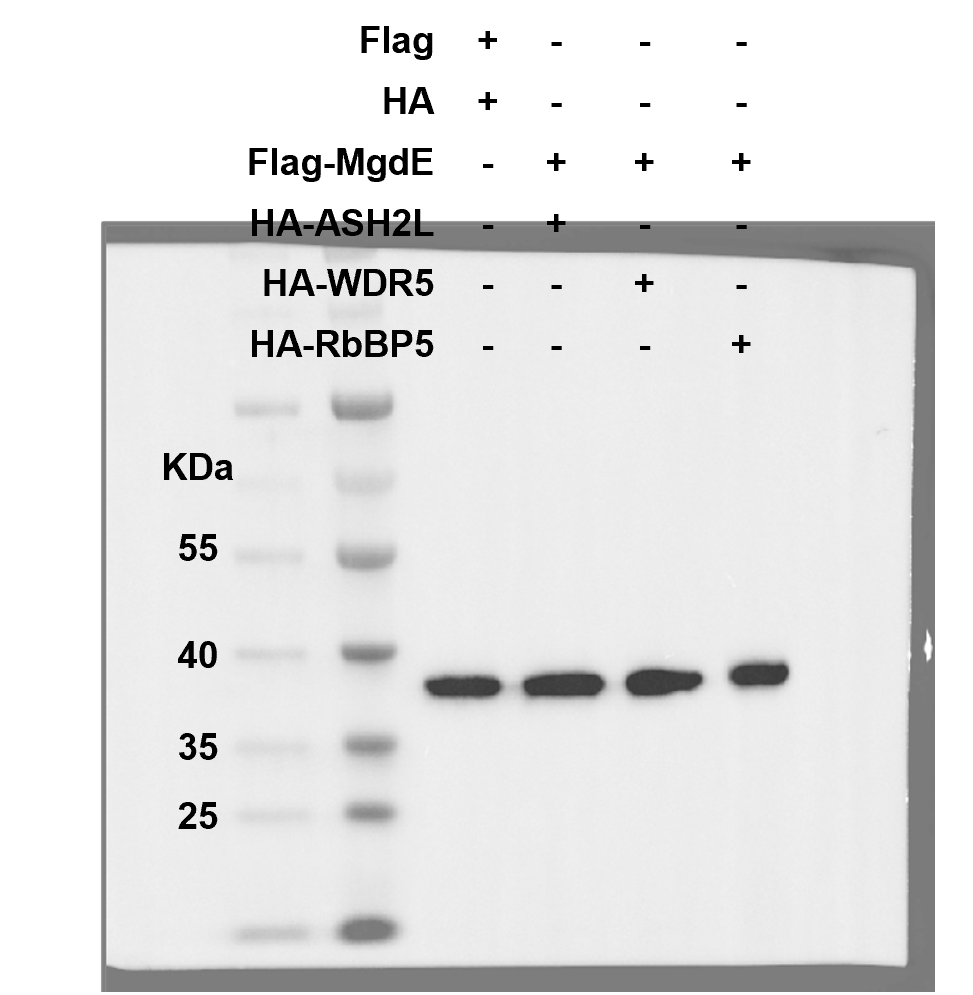

Supplement: Figure 4—source data 2. [file elife-107677-fig4-data2.zip › Figure 4B-Input-IB-GAPDH.tif]

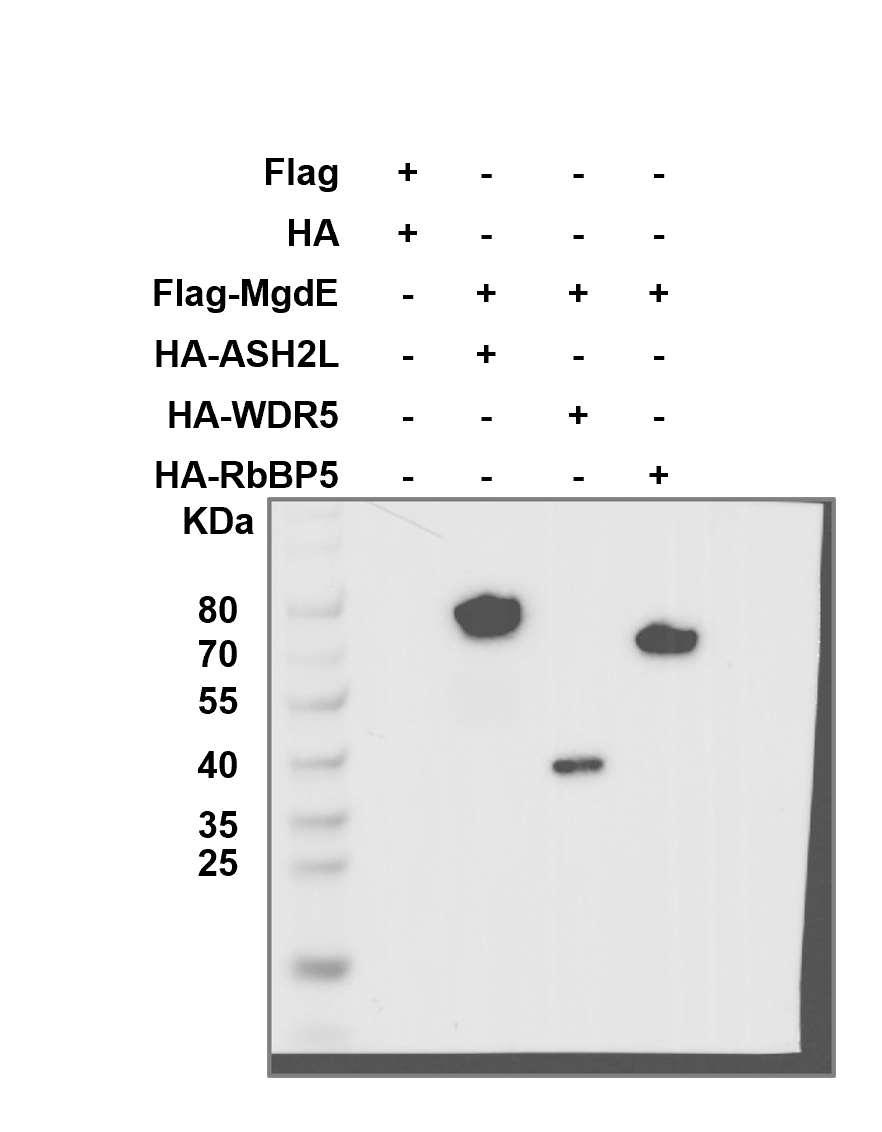

Supplement: Figure 4—source data 2. [file elife-107677-fig4-data2.zip › Figure 4B-Input-IB-HA.tif]

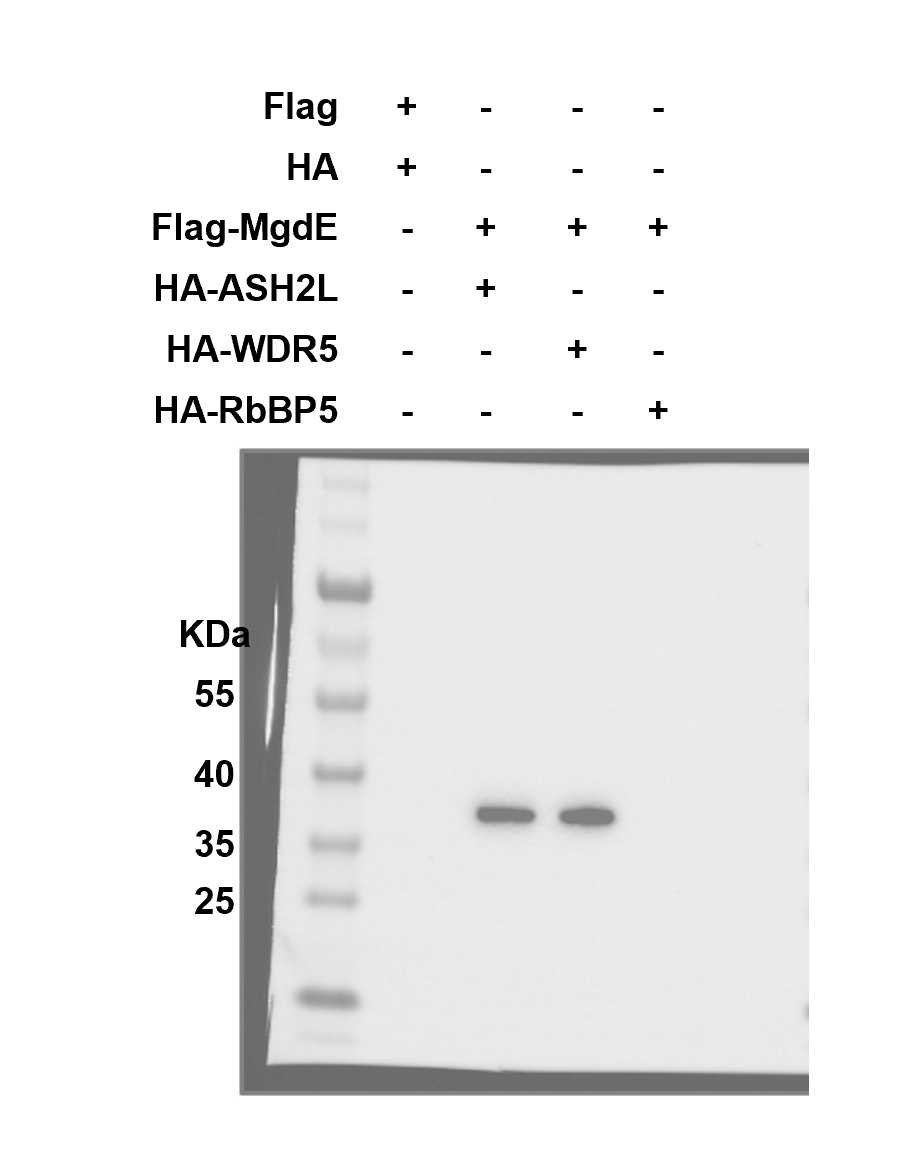

Supplement: Figure 4—source data 2. [file elife-107677-fig4-data2.zip › Figure 4B-IP-IB-Flag.tif]

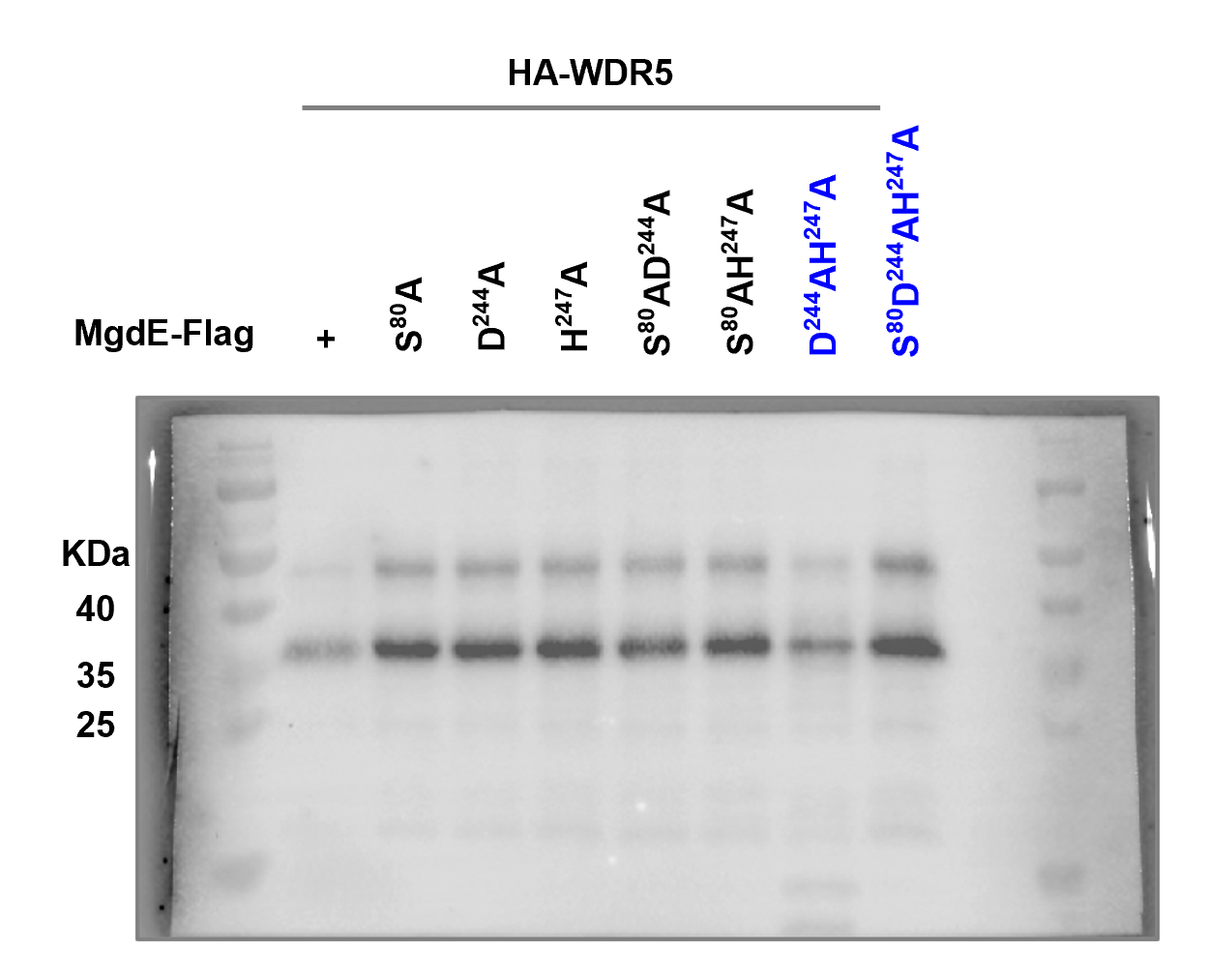

Supplement: Figure 4—source data 2. [file elife-107677-fig4-data2.zip › Figure 4B-IP-IB-HA.tif]

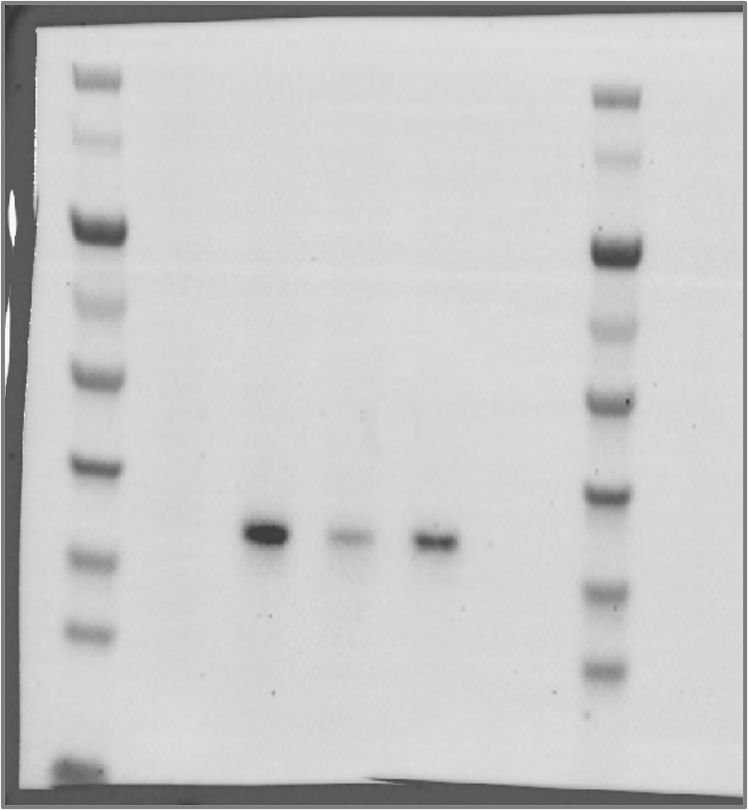

Supplement: Figure 4—source data 3. [file elife-107677-fig4-data3.zip › Figure 4—source data 3/Figure 4C-Input-IB-Flag.tif]

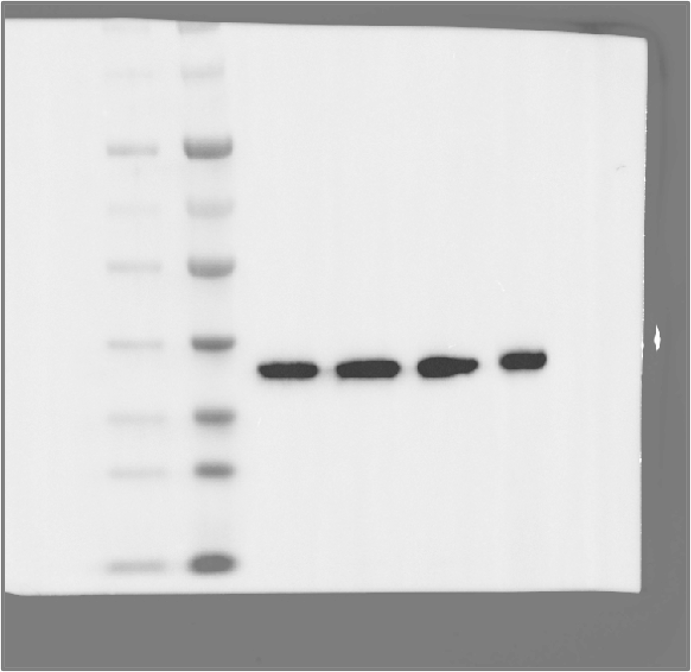

Supplement: Figure 4—source data 3. [file elife-107677-fig4-data3.zip › Figure 4—source data 3/Figure 4C-Input-IB-GAPDH.tif]

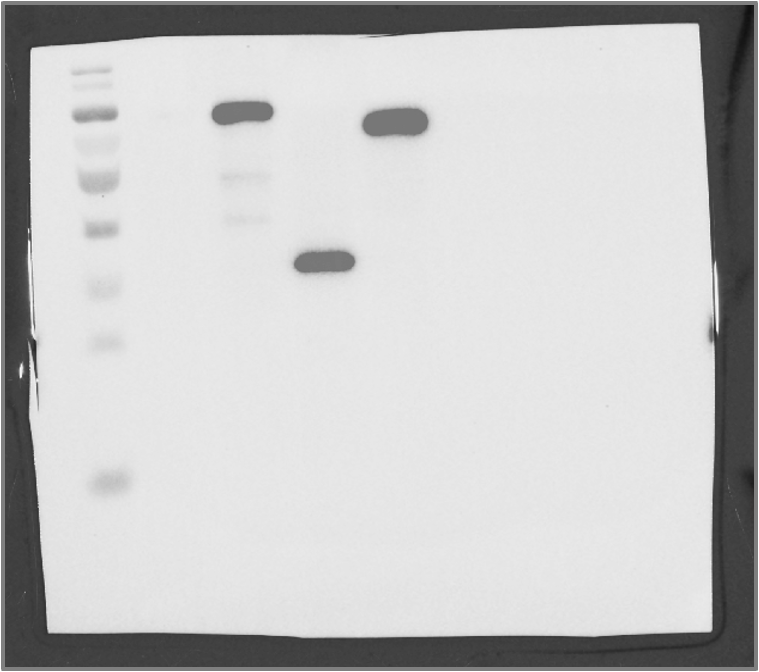

Supplement: Figure 4—source data 3. [file elife-107677-fig4-data3.zip › Figure 4—source data 3/Figure 4C-Input-IB-HA.tif]

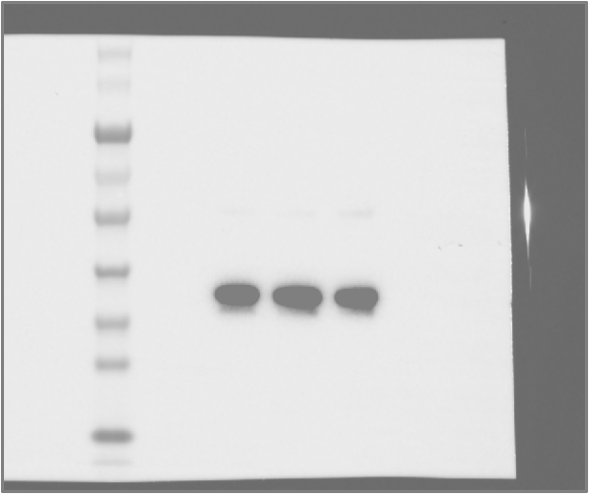

Supplement: Figure 4—source data 3. [file elife-107677-fig4-data3.zip › Figure 4—source data 3/Figure 4C-IP-IB-Flag.tif]

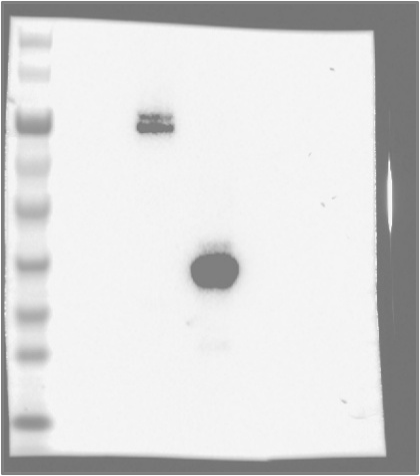

Supplement: Figure 4—source data 3. [file elife-107677-fig4-data3.zip › Figure 4—source data 3/Figure 4C-IP-IB-HA.tif]

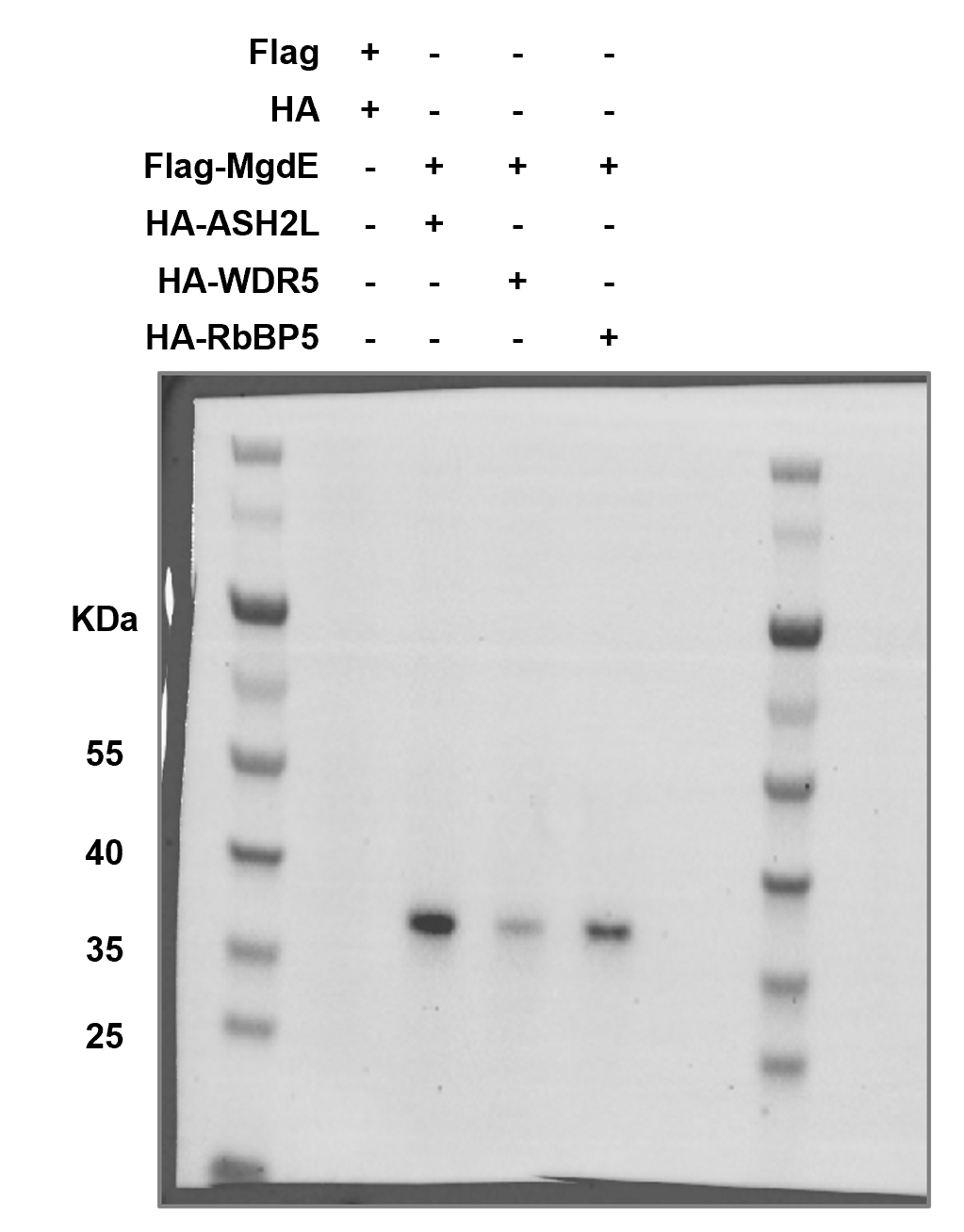

Supplement: Figure 4—source data 4. [file elife-107677-fig4-data4.zip › Figure 4—source data 4/Figure 4C-Input-IB-Flag.tif]

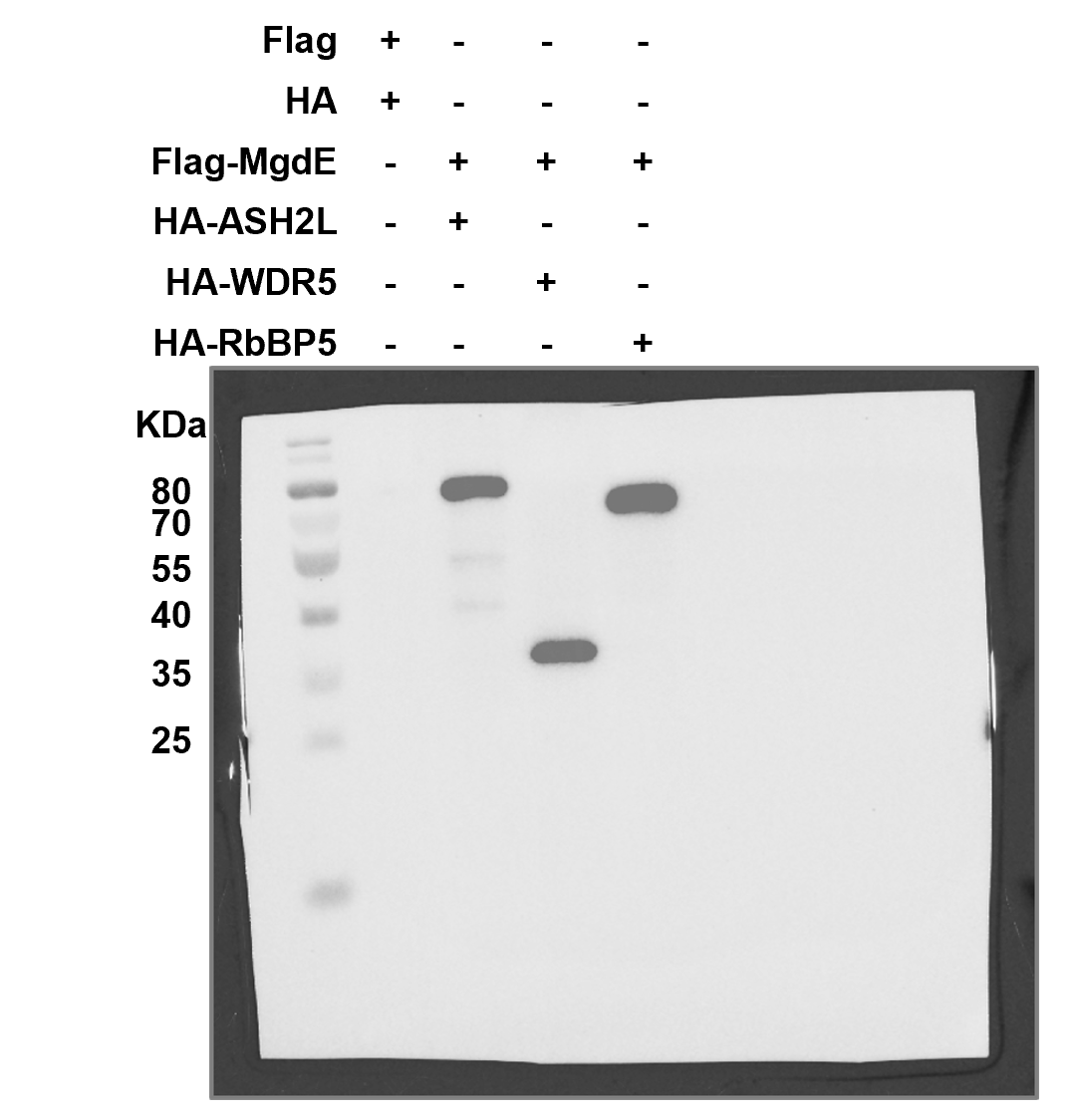

Supplement: Figure 4—source data 4. [file elife-107677-fig4-data4.zip › Figure 4—source data 4/Figure 4C-Input-IB-HA.tif]

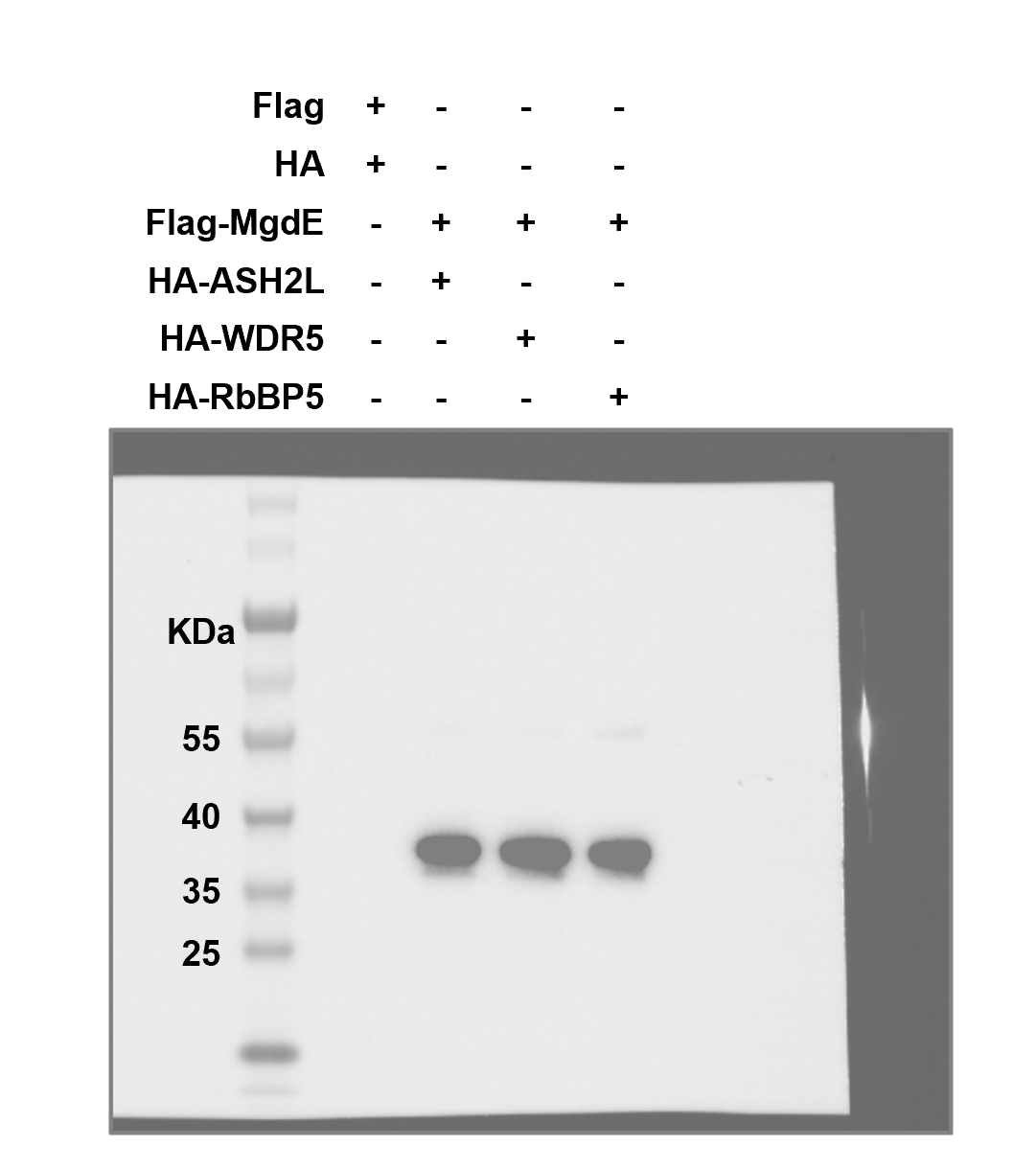

Supplement: Figure 4—source data 4. [file elife-107677-fig4-data4.zip › Figure 4—source data 4/Figure 4C-IP-IB-Flag.tif]

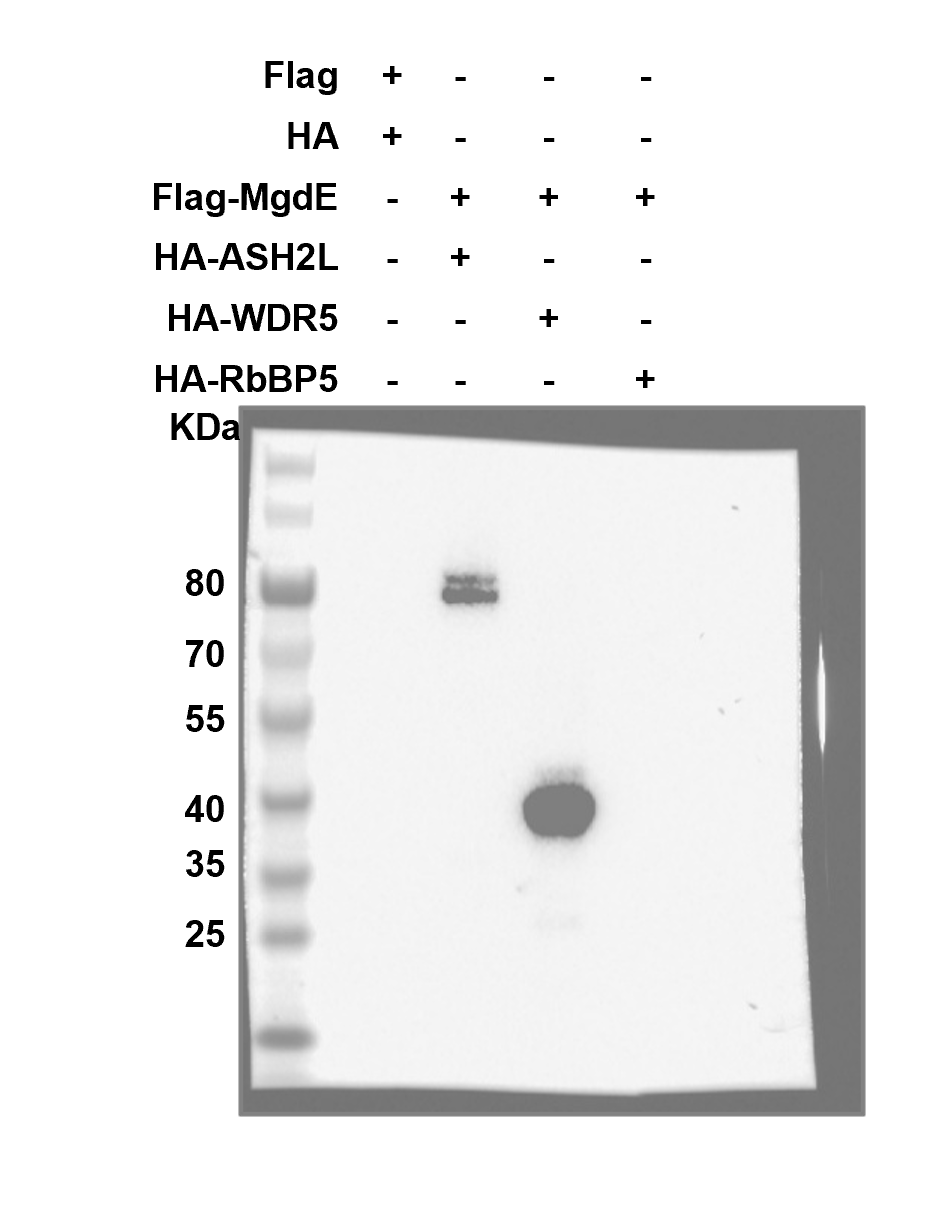

Supplement: Figure 4—source data 4. [file elife-107677-fig4-data4.zip › Figure 4—source data 4/Figure 4C-IP-IB-HA.tif]

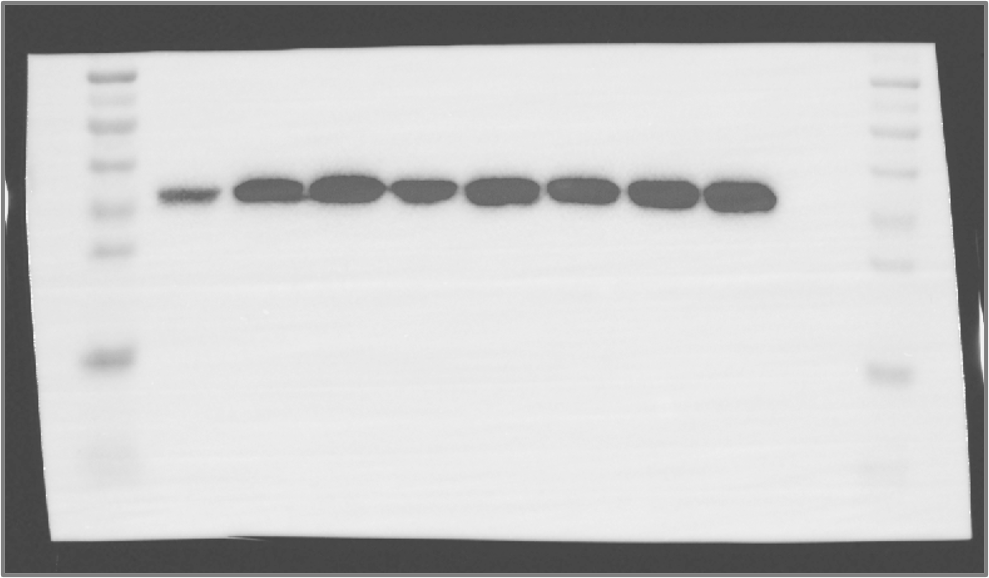

Supplement: Figure 4—figure supplement 1—source data 1. [file elife-107677-fig4-figsupp1-data1.zip › Figure4-figure supplement—source data 1/Figure4-figure supplement 4C-Input-IB-Flag.tif]

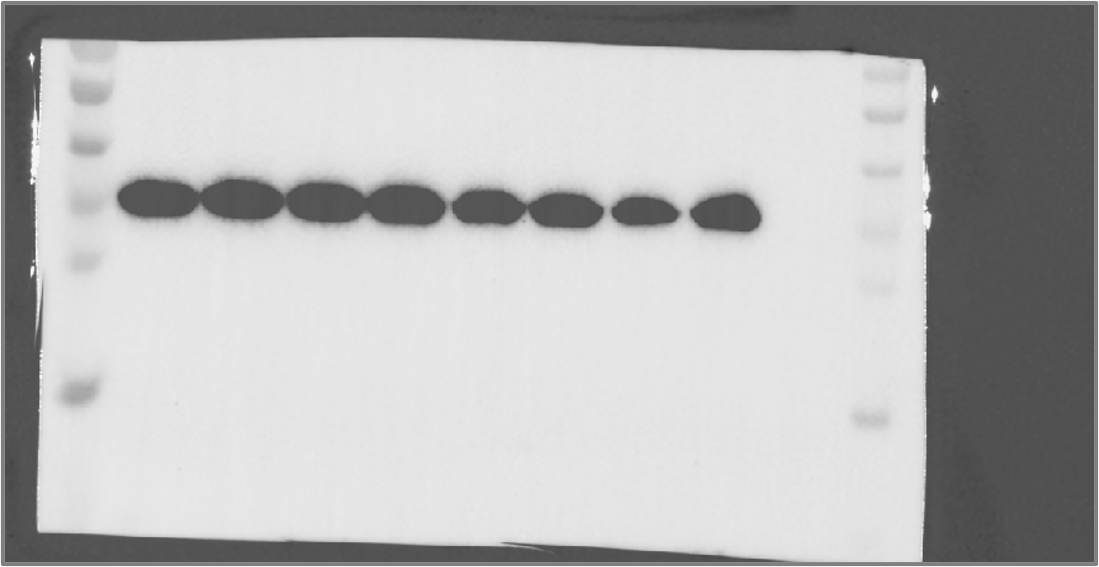

Supplement: Figure 4—figure supplement 1—source data 1. [file elife-107677-fig4-figsupp1-data1.zip › Figure4-figure supplement—source data 1/Figure4-figure supplement 4C-Input-IB-GAPDH.tif]

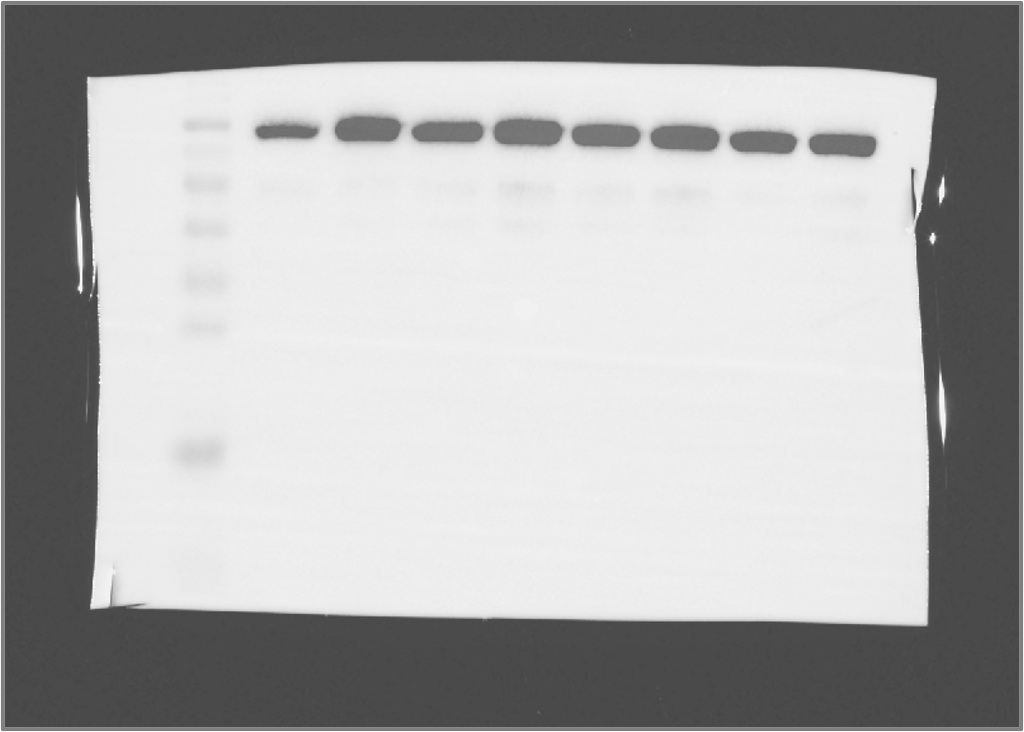

Supplement: Figure 4—figure supplement 1—source data 1. [file elife-107677-fig4-figsupp1-data1.zip › Figure4-figure supplement—source data 1/Figure4-figure supplement 4C-Input-IB-HA.tif]

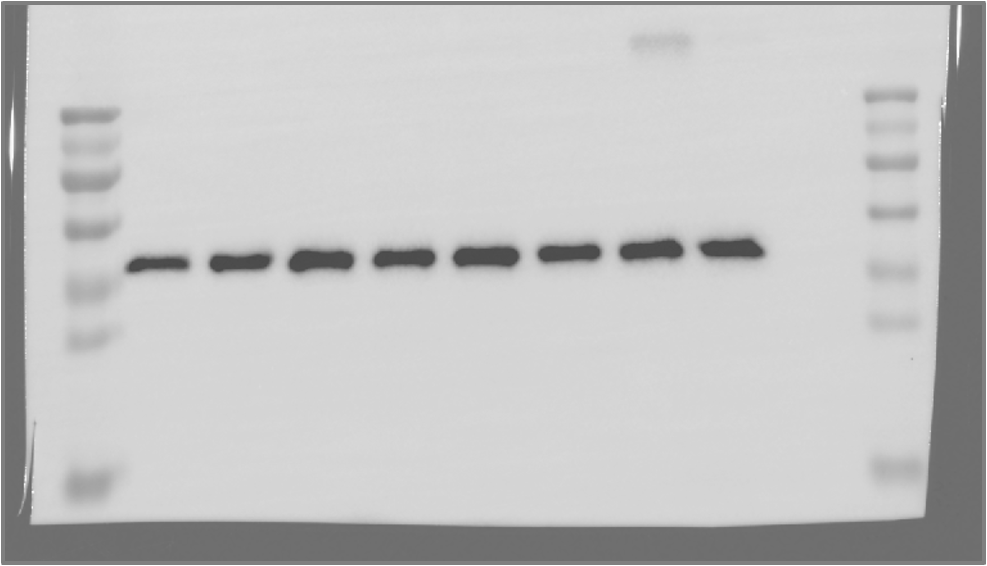

Supplement: Figure 4—figure supplement 1—source data 1. [file elife-107677-fig4-figsupp1-data1.zip › Figure4-figure supplement—source data 1/Figure4-figure supplement 4C-IP-IB-Flag.tif]

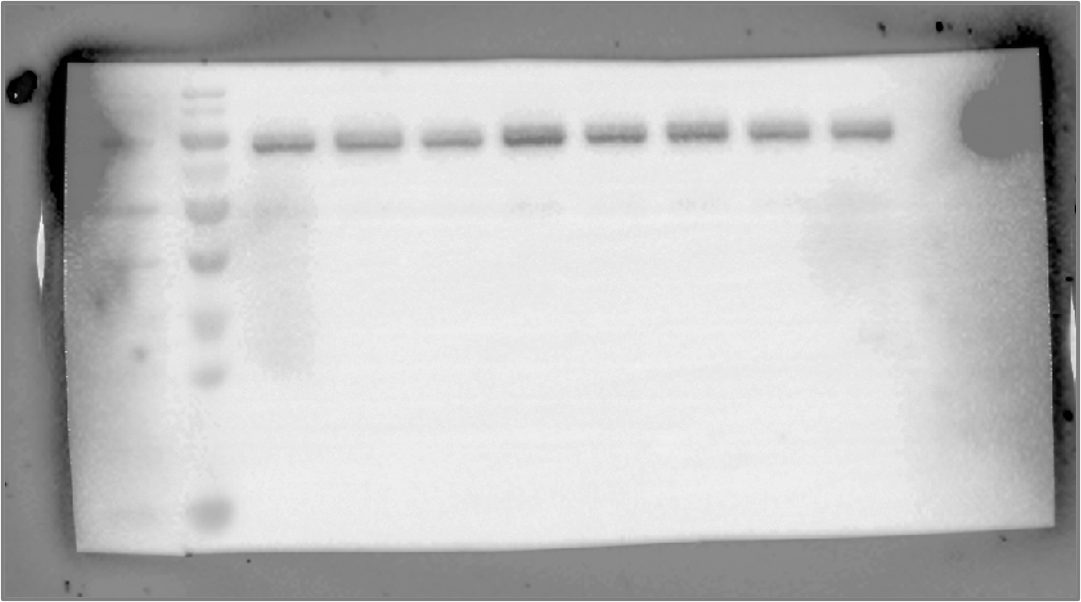

Supplement: Figure 4—figure supplement 1—source data 1. [file elife-107677-fig4-figsupp1-data1.zip › Figure4-figure supplement—source data 1/Figure4-figure supplement 4C-IP-IB-HA.tif]

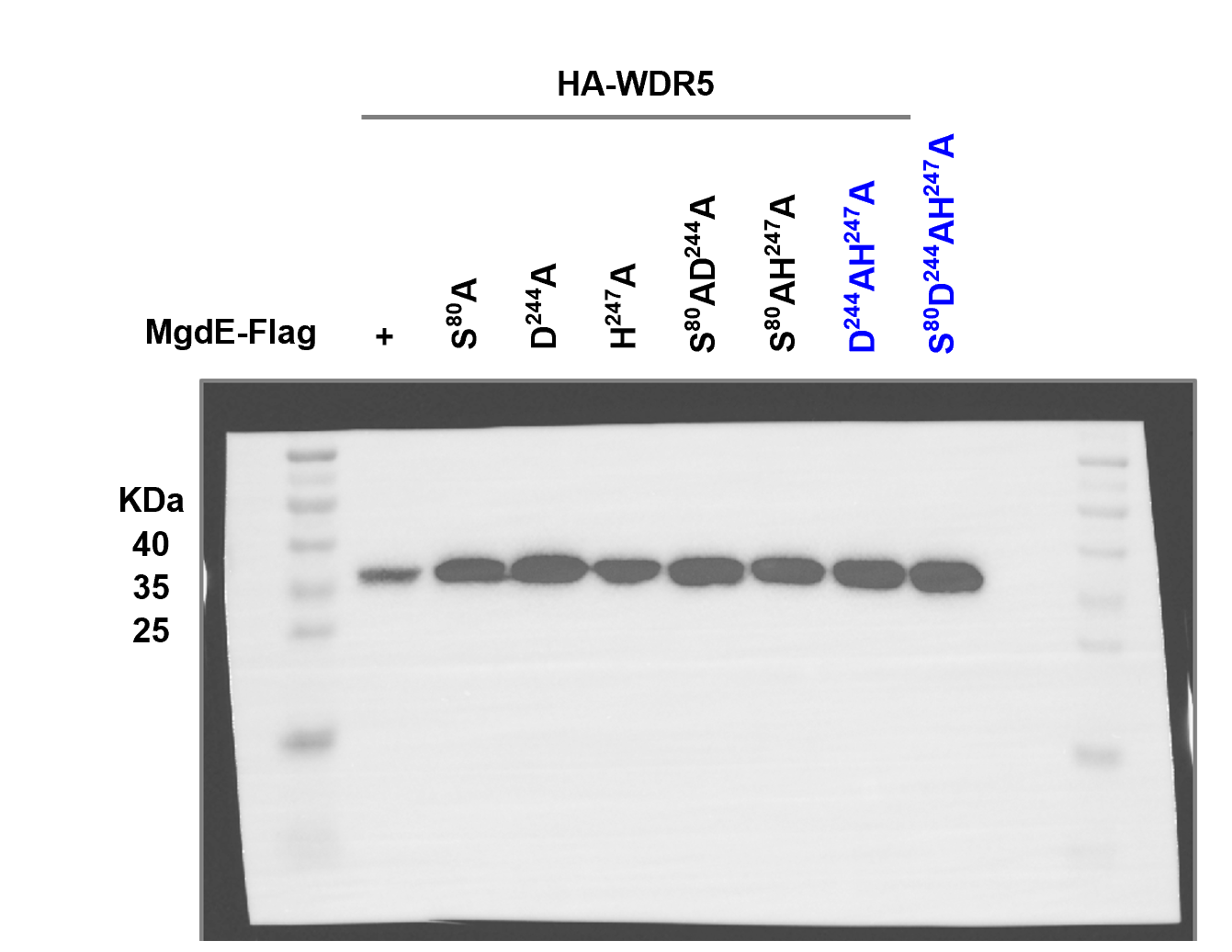

Supplement: Figure 4—figure supplement 1—source data 2. [file elife-107677-fig4-figsupp1-data2.zip › Figure4-figure supplement—source data 2/Figure4-figure supplement 4C-Input-IB-Flag.tif]

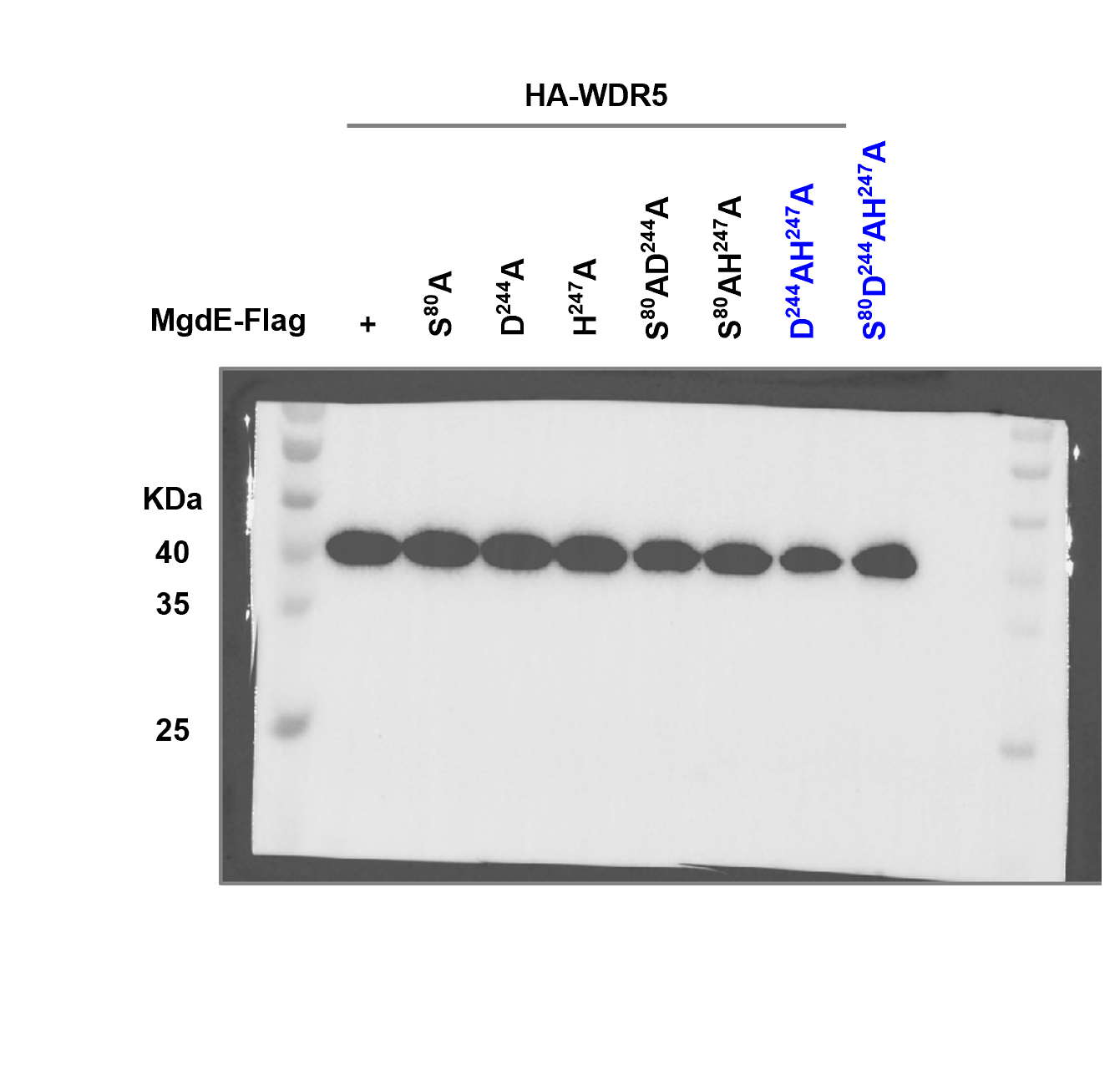

Supplement: Figure 4—figure supplement 1—source data 2. [file elife-107677-fig4-figsupp1-data2.zip › Figure4-figure supplement—source data 2/Figure4-figure supplement 4C-Input-IB-GAPDH.tif]

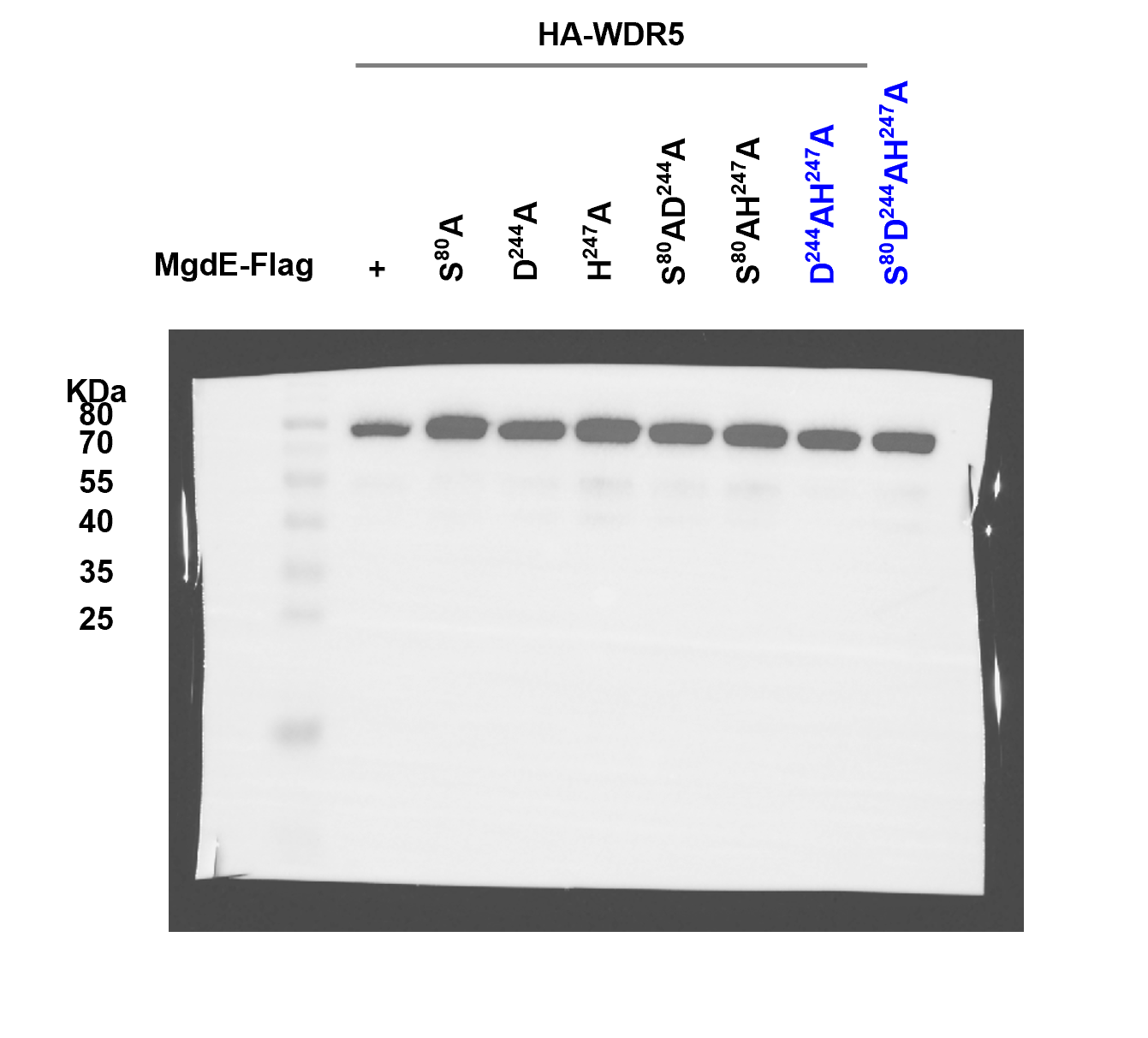

Supplement: Figure 4—figure supplement 1—source data 2. [file elife-107677-fig4-figsupp1-data2.zip › Figure4-figure supplement—source data 2/Figure4-figure supplement 4C-Input-IB-HA.tif]

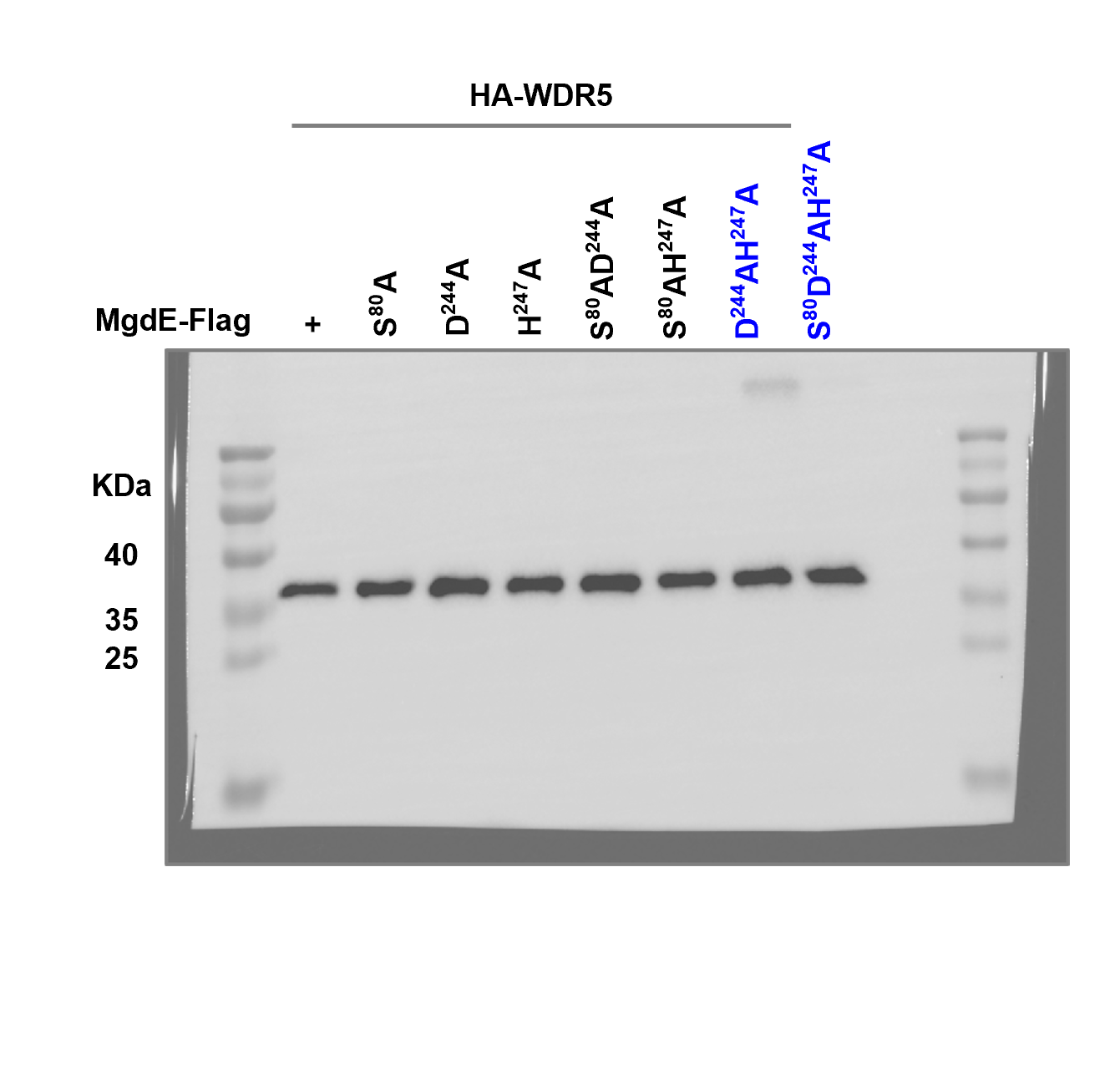

Supplement: Figure 4—figure supplement 1—source data 2. [file elife-107677-fig4-figsupp1-data2.zip › Figure4-figure supplement—source data 2/Figure4-figure supplement 4C-IP-IB-Flag.tif]

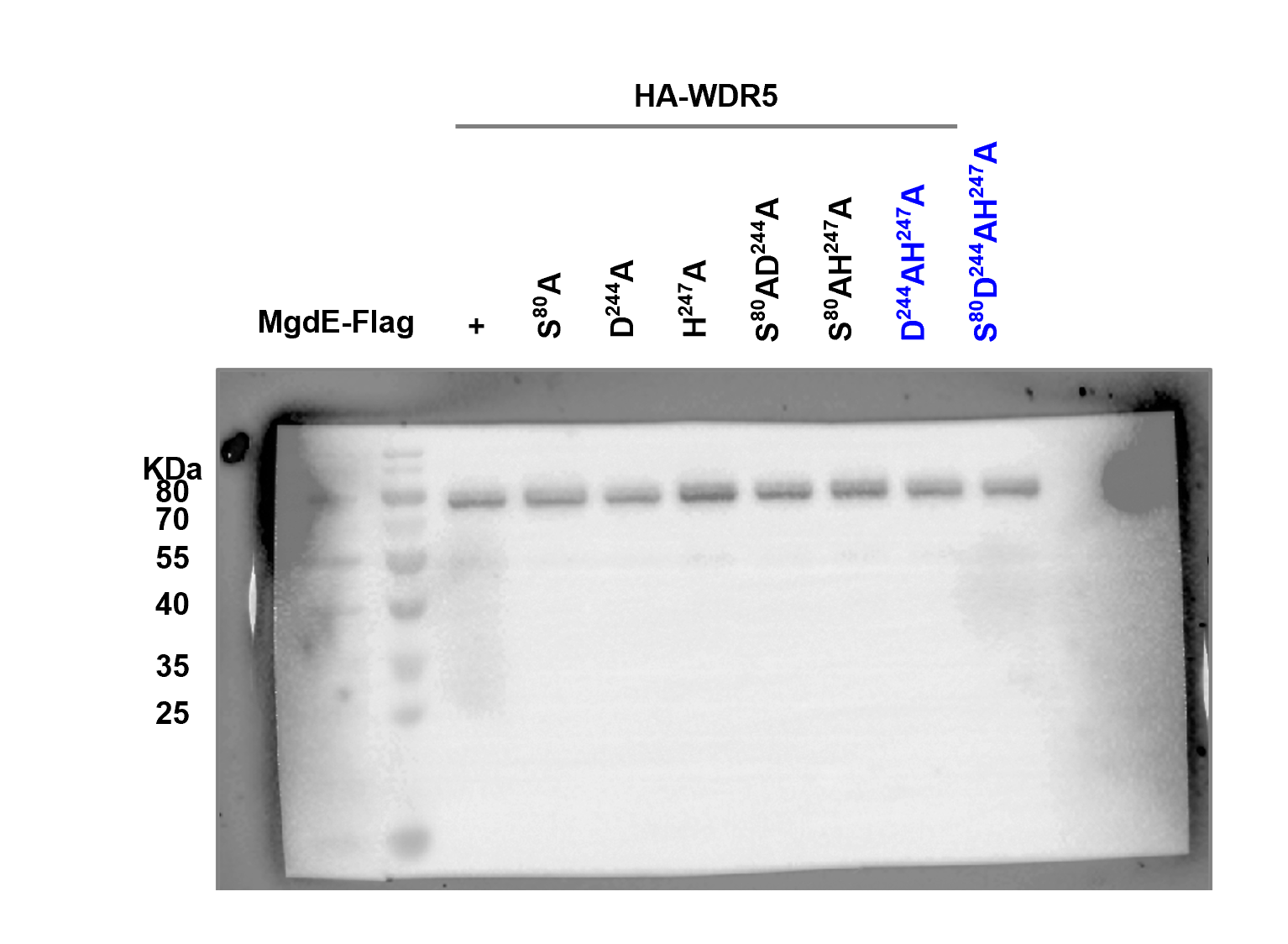

Supplement: Figure 4—figure supplement 1—source data 2. [file elife-107677-fig4-figsupp1-data2.zip › Figure4-figure supplement—source data 2/Figure4-figure supplement 4C-IP-IB-HA.tif]

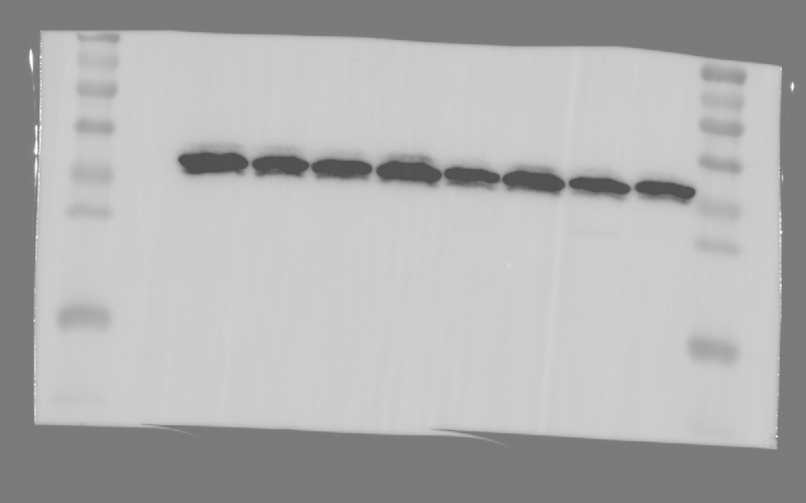

Supplement: Figure 5—source data 1. [file elife-107677-fig5-data1.zip › Figure 5B-Input-IB-Flag.tif]

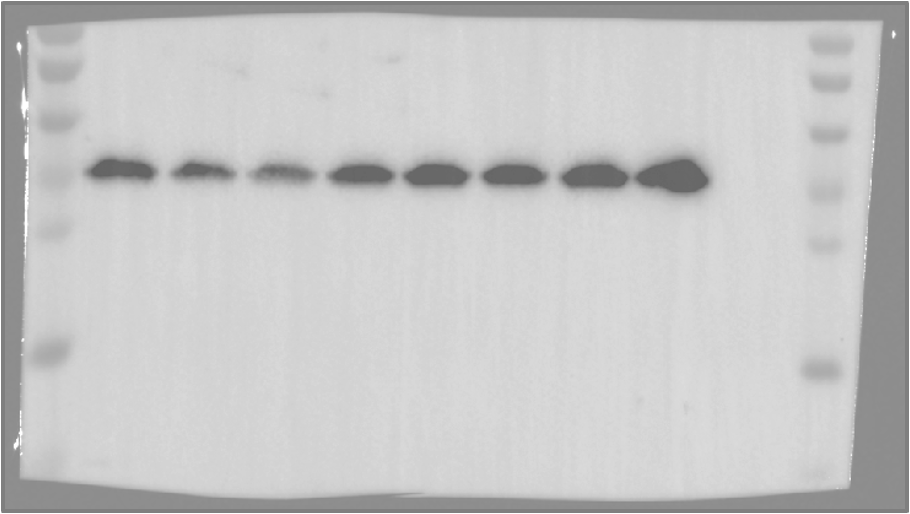

Supplement: Figure 5—source data 1. [file elife-107677-fig5-data1.zip › Figure 5B-Input-IB-GAPDH.tif]

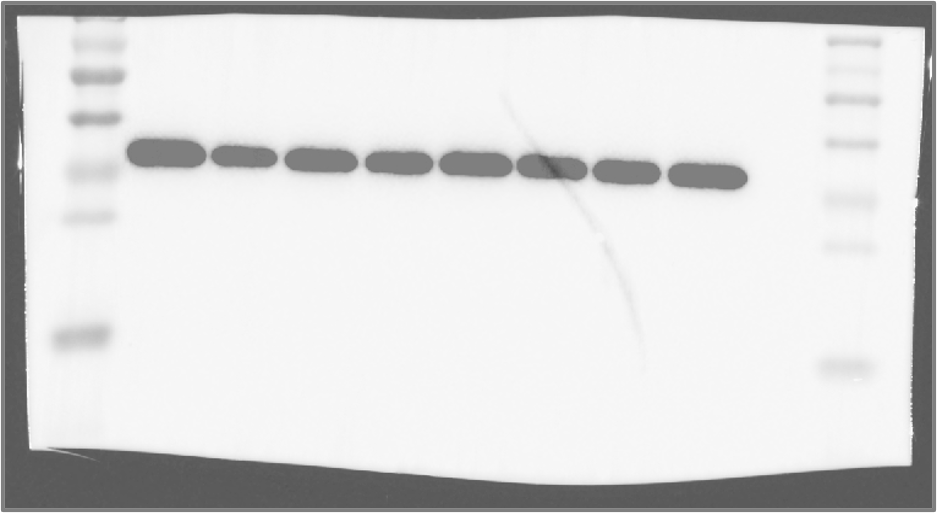

Supplement: Figure 5—source data 1. [file elife-107677-fig5-data1.zip › Figure 5B-Input-IB-HA.tif]

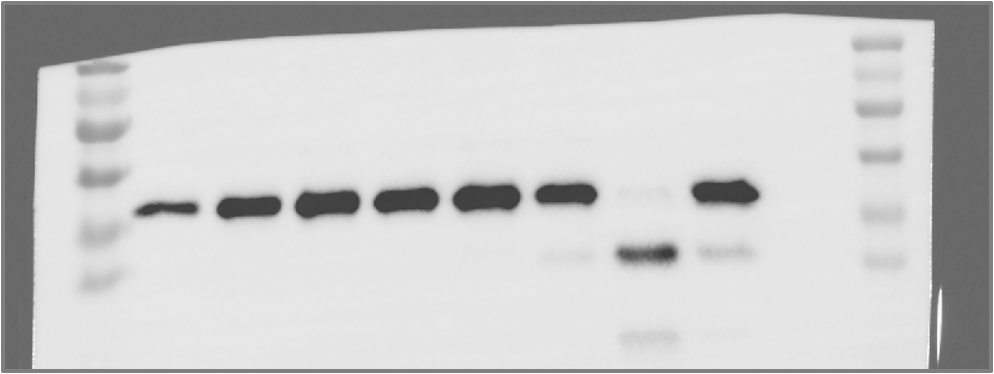

Supplement: Figure 5—source data 1. [file elife-107677-fig5-data1.zip › Figure 5B-IP-IB-Flag.tif]

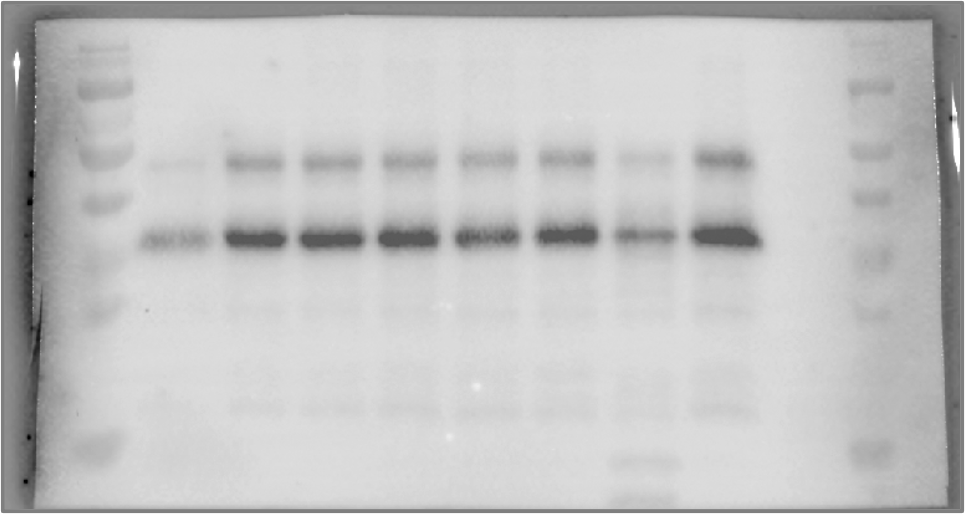

Supplement: Figure 5—source data 1. [file elife-107677-fig5-data1.zip › Figure 5B-IP-IB-HA.tif]

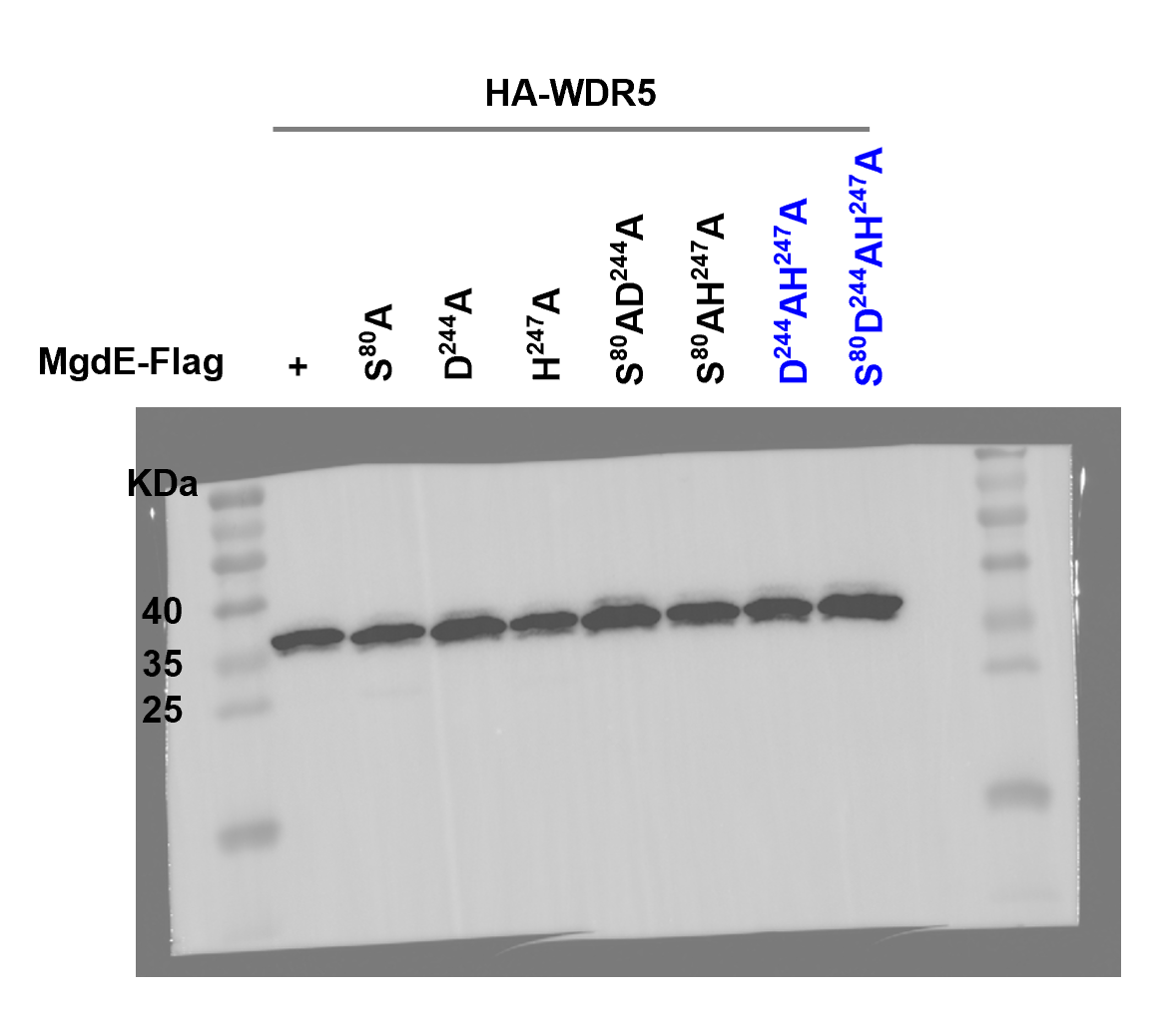

Supplement: Figure 5—source data 2. [file elife-107677-fig5-data2.zip › Figure 5B-Input-IB-Flag.tif]

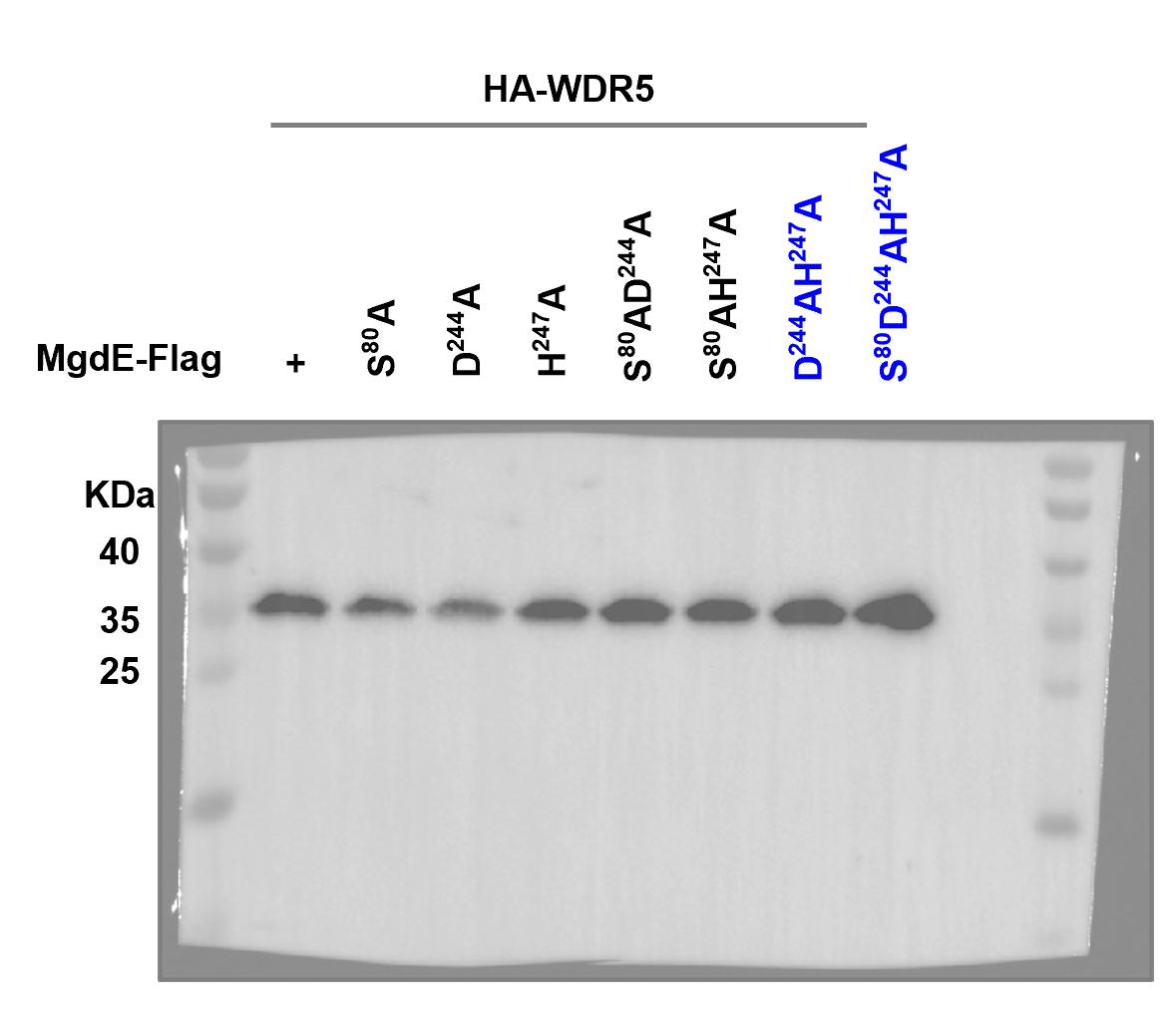

Supplement: Figure 5—source data 2. [file elife-107677-fig5-data2.zip › Figure 5B-Input-IB-GAPDH.tif]

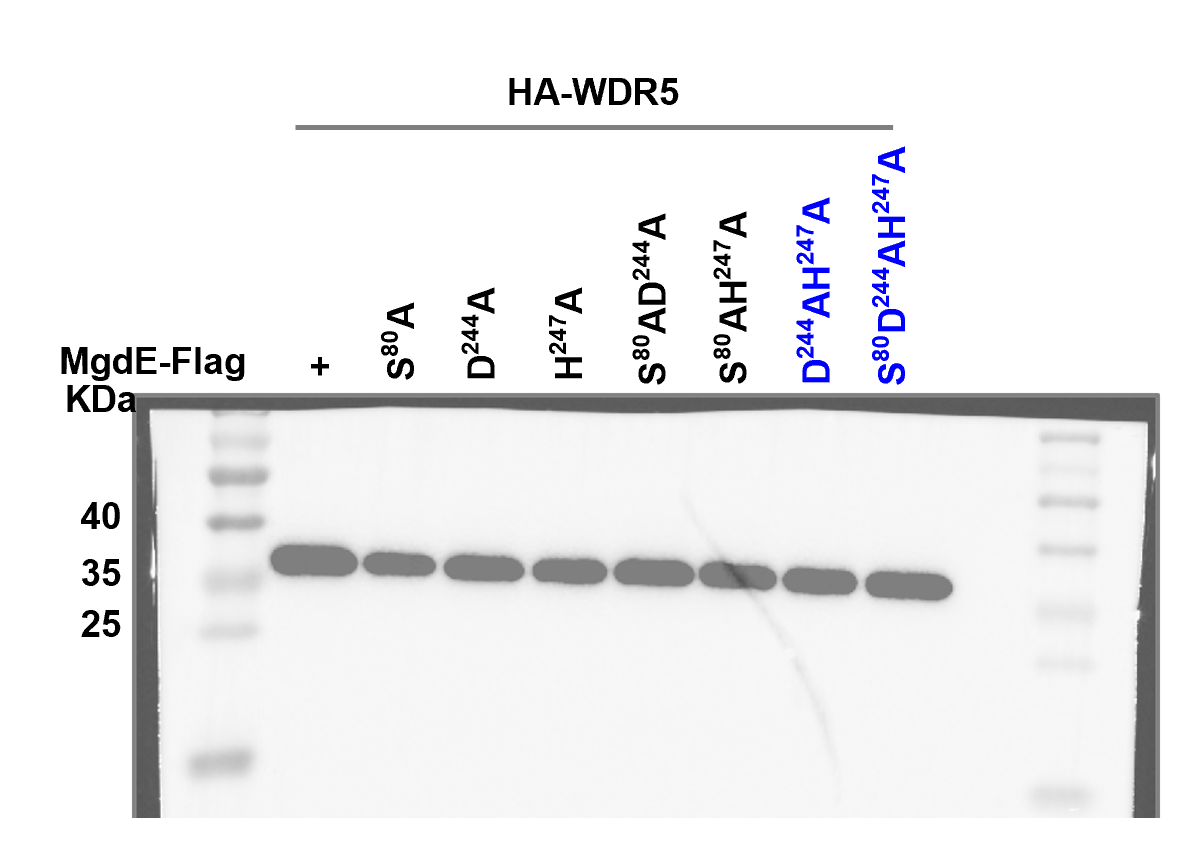

Supplement: Figure 5—source data 2. [file elife-107677-fig5-data2.zip › Figure 5B-Input-IB-HA.tif]

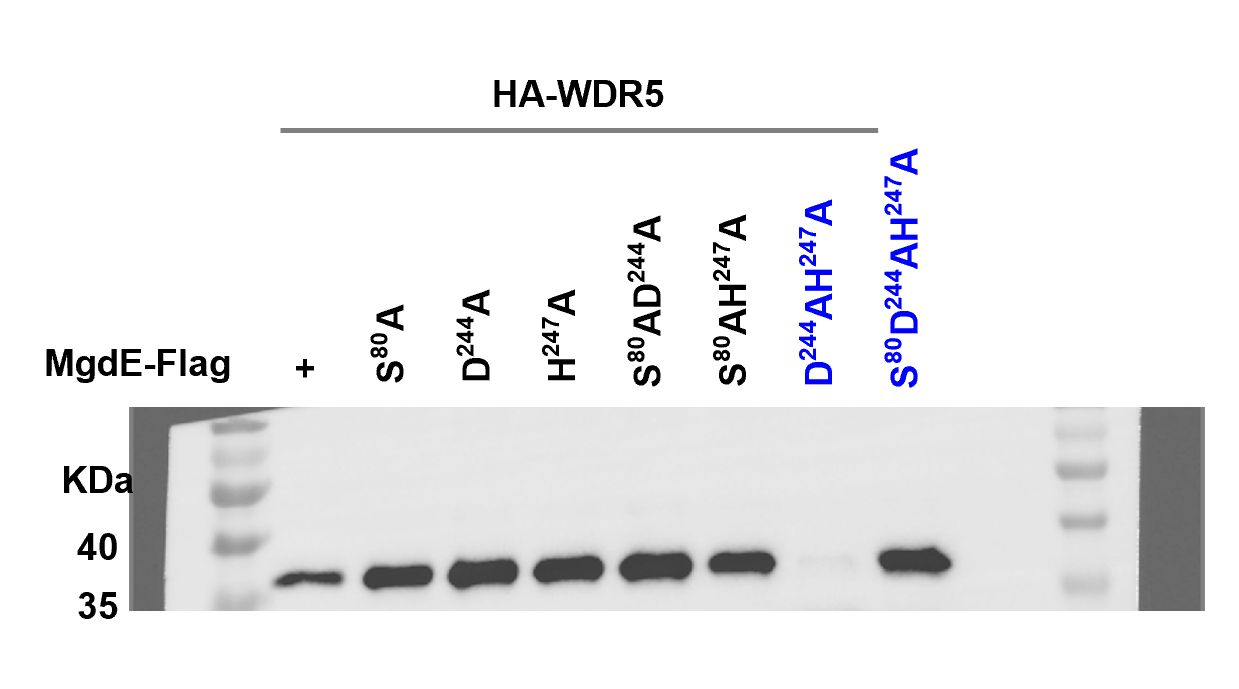

Supplement: Figure 5—source data 2. [file elife-107677-fig5-data2.zip › Figure 5B-IP-IB-Flag.tif]

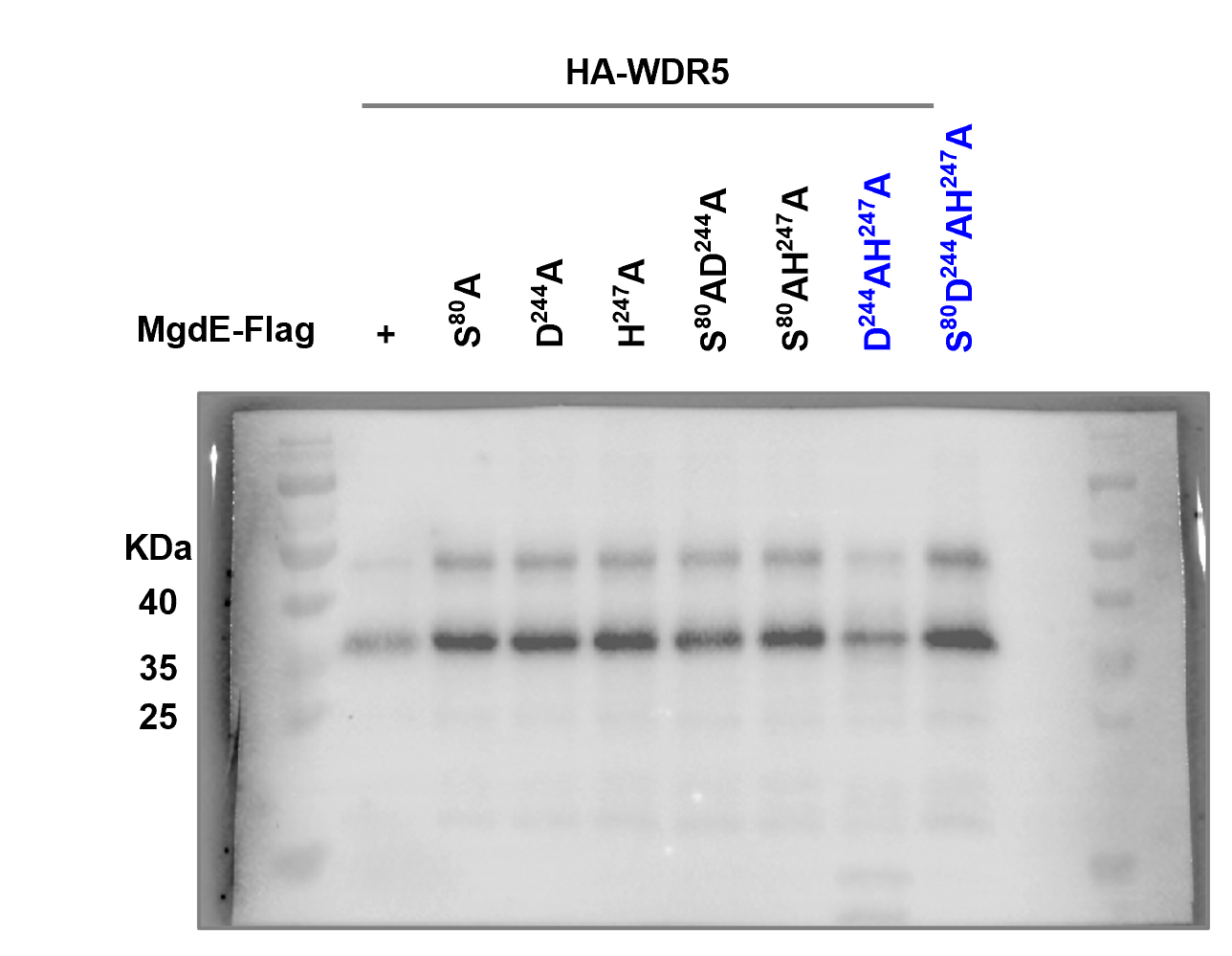

Supplement: Figure 5—source data 2. [file elife-107677-fig5-data2.zip › Figure 5B-IP-IB-HA.tif]

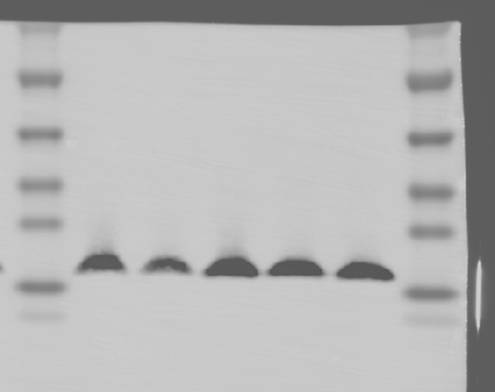

Supplement: Figure 5—source data 3. [file elife-107677-fig5-data3.zip › Figure 5—source data 3/Figure 5D-EGFP-H3.tif]

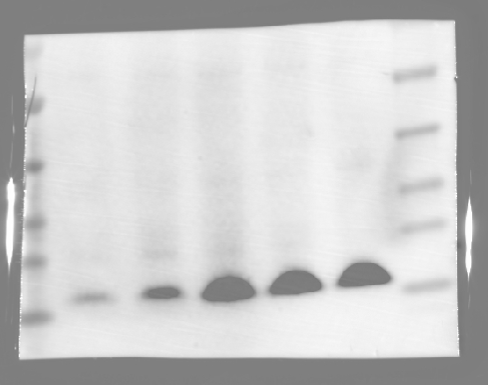

Supplement: Figure 5—source data 3. [file elife-107677-fig5-data3.zip › Figure 5—source data 3/Figure 5D-EGFP-H3K4me3-1.tif]

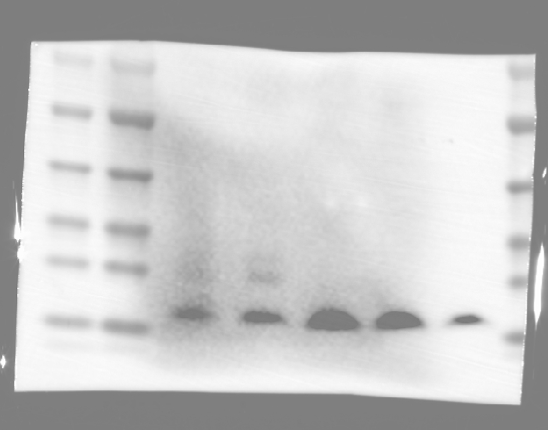

Supplement: Figure 5—source data 3. [file elife-107677-fig5-data3.zip › Figure 5—source data 3/Figure 5D-EGFP-H3K4me3-2.tif]

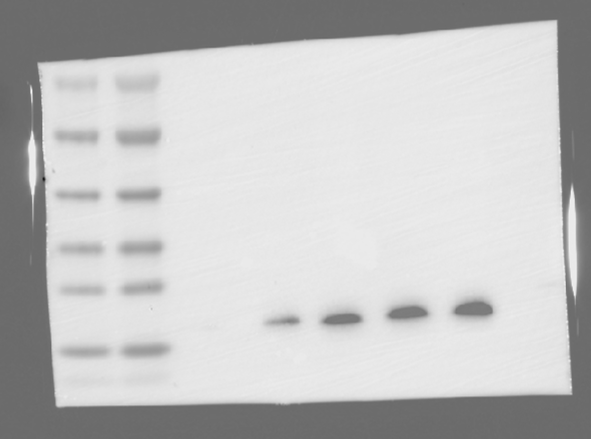

Supplement: Figure 5—source data 3. [file elife-107677-fig5-data3.zip › Figure 5—source data 3/Figure 5D-EGFP-H3K4me3-3.tif]

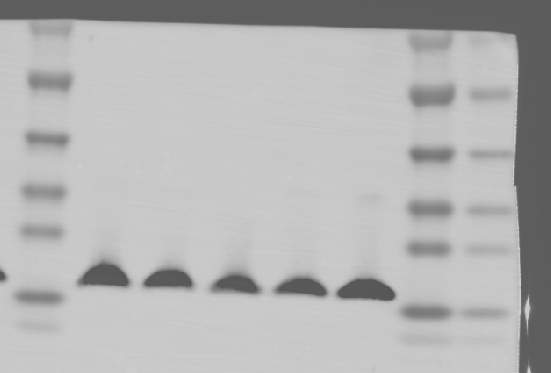

Supplement: Figure 5—source data 3. [file elife-107677-fig5-data3.zip › Figure 5—source data 3/Figure 5D-MgdE-D244AH247A-EGFP-H3.tif]

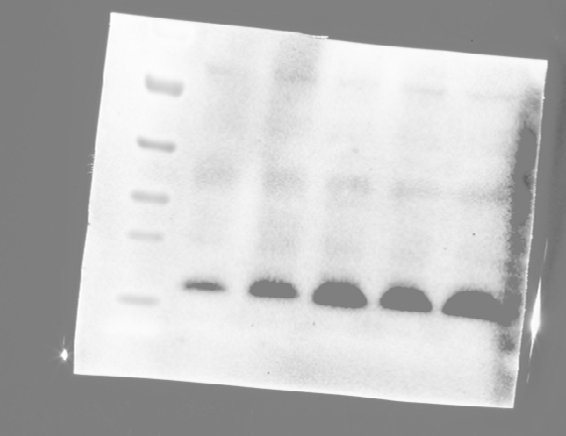

Supplement: Figure 5—source data 3. [file elife-107677-fig5-data3.zip › Figure 5—source data 3/Figure 5D-MgdE-D244AH247A-EGFP-H3K4me3-1.tif]

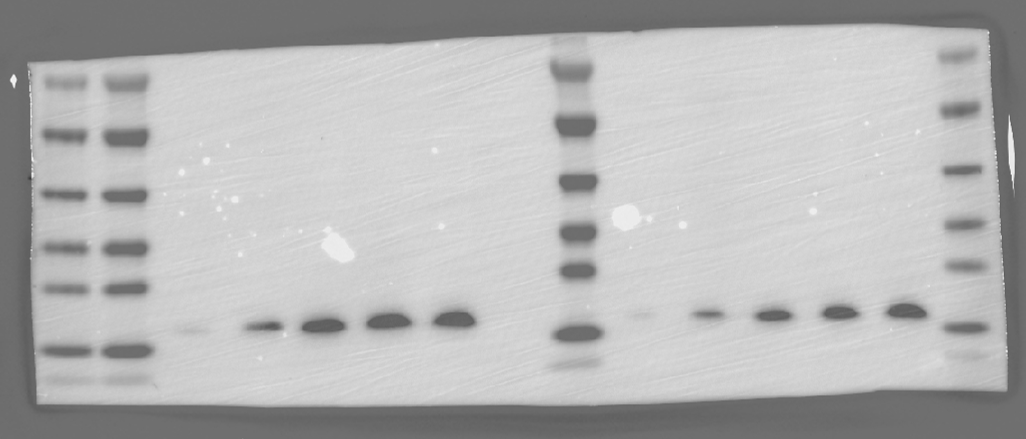

Supplement: Figure 5—source data 3. [file elife-107677-fig5-data3.zip › Figure 5—source data 3/Figure 5D-MgdE-D244AH247A-EGFP-H3K4me3-2 and 3.tif]

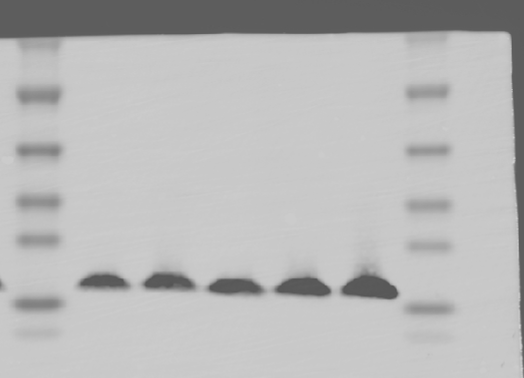

Supplement: Figure 5—source data 3. [file elife-107677-fig5-data3.zip › Figure 5—source data 3/Figure 5D-MgdE-EGFP-H3-1.tif]

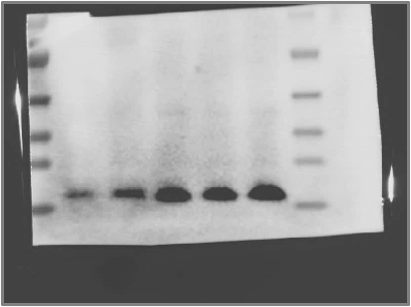

Supplement: Figure 5—source data 3. [file elife-107677-fig5-data3.zip › Figure 5—source data 3/Figure 5D-MgdE-EGFP-H3K4me3-1.tif]

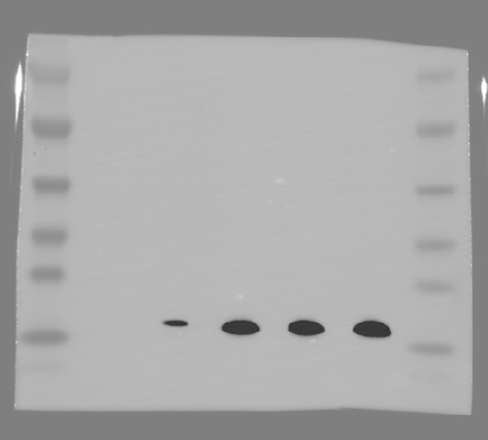

Supplement: Figure 5—source data 3. [file elife-107677-fig5-data3.zip › Figure 5—source data 3/Figure 5D-MgdE-EGFP-H3K4me3-2.tif]

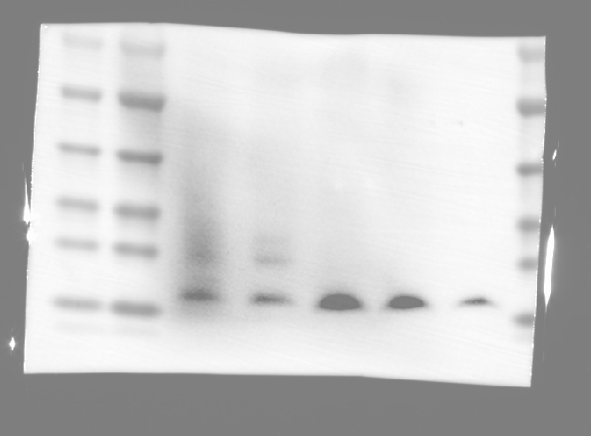

Supplement: Figure 5—source data 3. [file elife-107677-fig5-data3.zip › Figure 5—source data 3/Figure 5D-MgdE-EGFP-H3K4me3-3.tif]

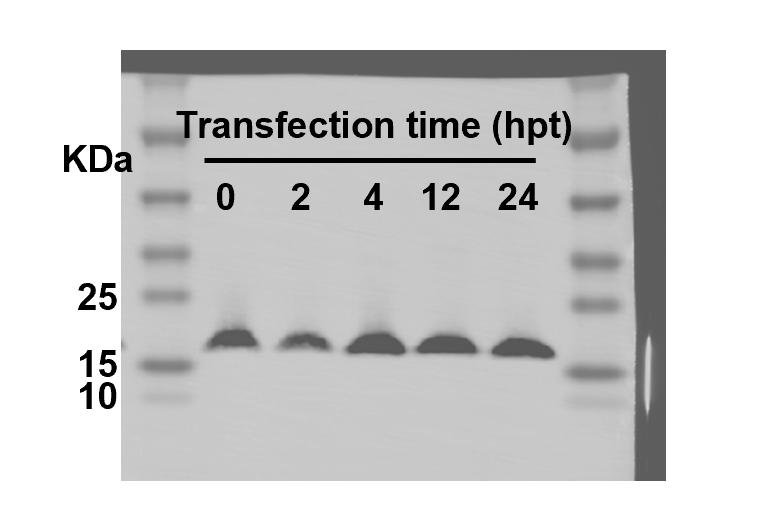

Supplement: Figure 5—source data 4. [file elife-107677-fig5-data4.zip › Figure 5—source data 4/Figure 5D-EGFP-H3.tif]

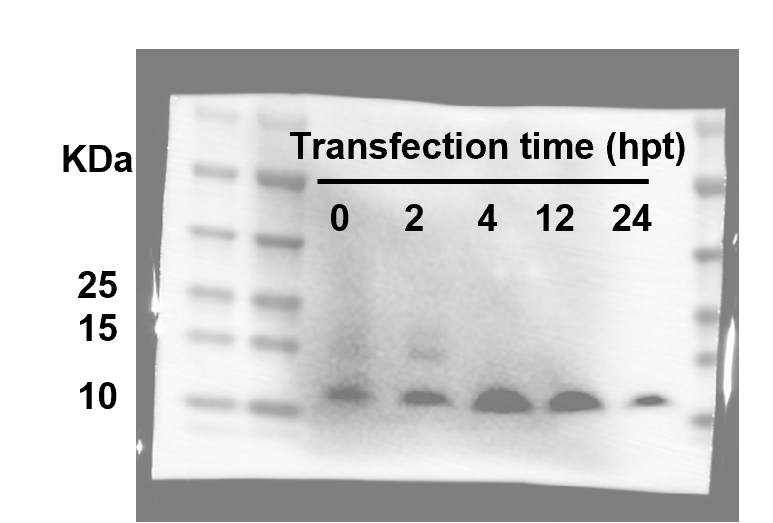

Supplement: Figure 5—source data 4. [file elife-107677-fig5-data4.zip › Figure 5—source data 4/Figure 5D-EGFP-H3K4me3-1.tif]

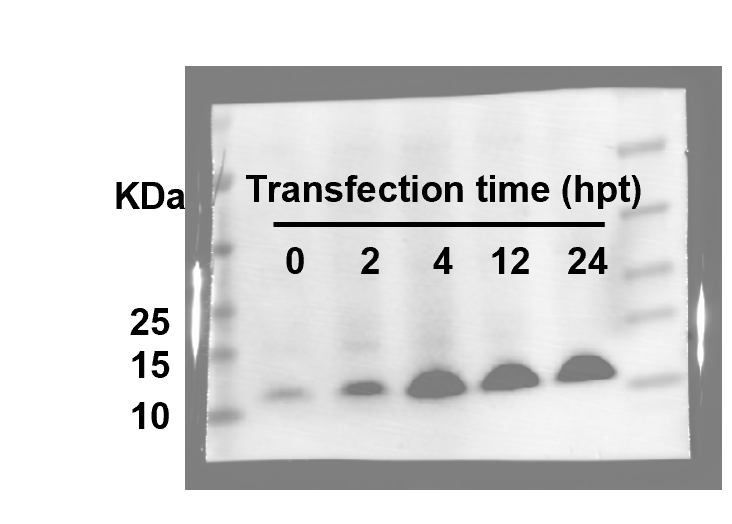

Supplement: Figure 5—source data 4. [file elife-107677-fig5-data4.zip › Figure 5—source data 4/Figure 5D-EGFP-H3K4me3-2.tif]

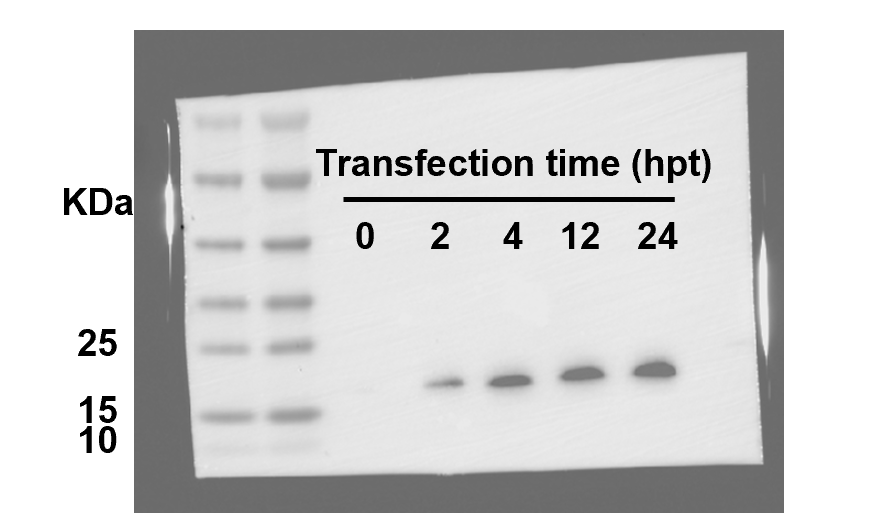

Supplement: Figure 5—source data 4. [file elife-107677-fig5-data4.zip › Figure 5—source data 4/Figure 5D-EGFP-H3K4me3-3.tif]

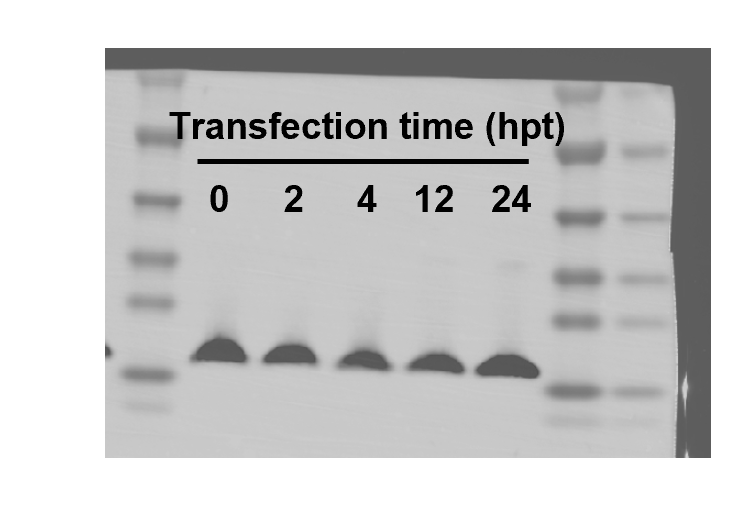

Supplement: Figure 5—source data 4. [file elife-107677-fig5-data4.zip › Figure 5—source data 4/Figure 5D-MgdE-D244AH247A-EGFP-H3.tif]

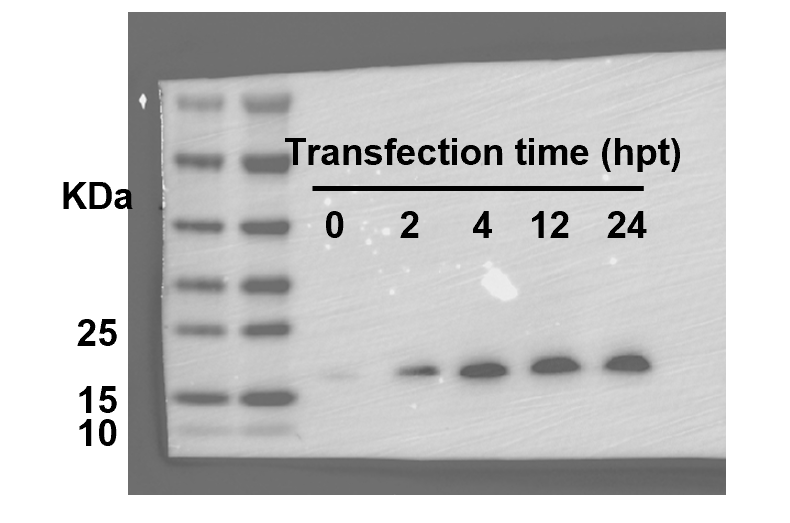

Supplement: Figure 5—source data 4. [file elife-107677-fig5-data4.zip › Figure 5—source data 4/Figure 5D-MgdE-D244AH247A-EGFP-H3K4me3-1.tif]

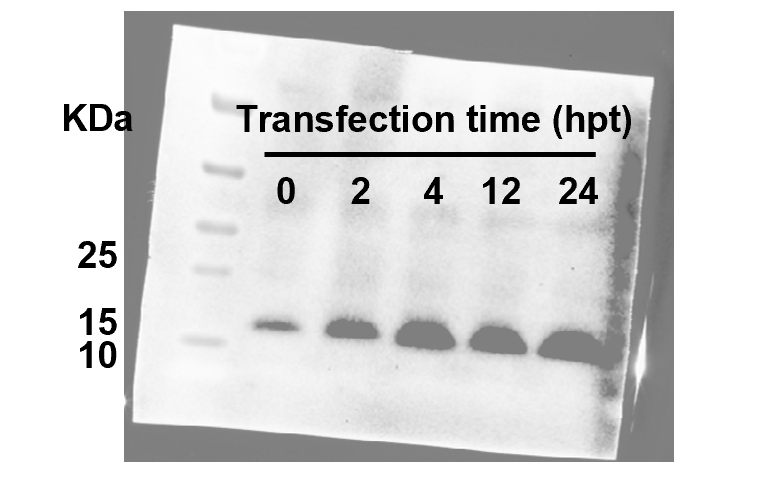

Supplement: Figure 5—source data 4. [file elife-107677-fig5-data4.zip › Figure 5—source data 4/Figure 5D-MgdE-D244AH247A-EGFP-H3K4me3-2.tif]

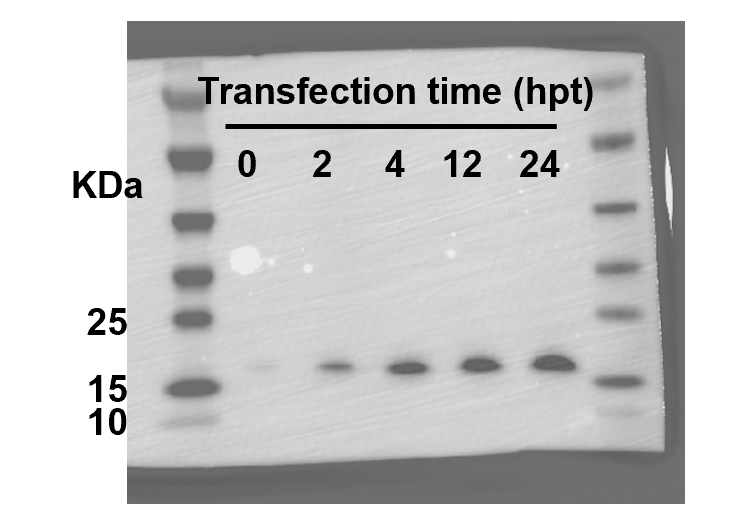

Supplement: Figure 5—source data 4. [file elife-107677-fig5-data4.zip › Figure 5—source data 4/Figure 5D-MgdE-D244AH247A-EGFP-H3K4me3-3.tif]
