# Supplementary figures and images for: A conserved mycobacterial nucleomodulin hijacks the host COMPASS complex to reprogram pro-inflammatory transcription and promote intracellular survival (part 2 of 2)
Source: eLife. 2026 Mar 31;14:RP107677. doi: 10.7554/eLife.107677 (PMC13038263; doi:10.7554/eLife.107677)

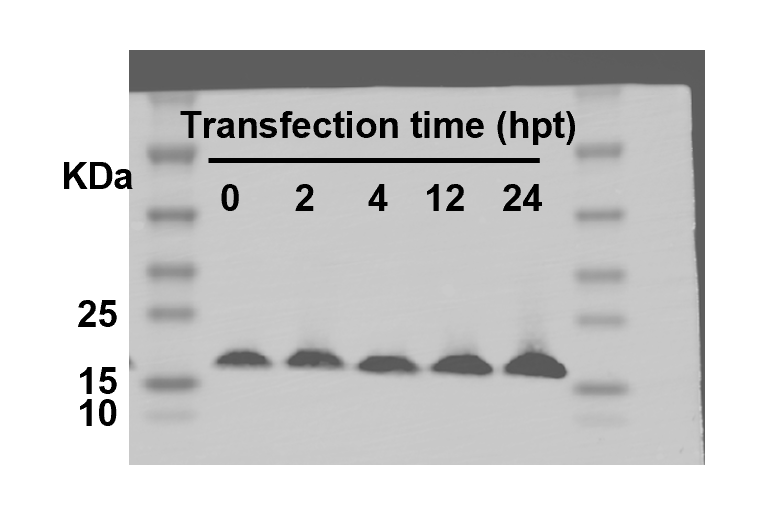

Supplement: Figure 5—source data 4. [file elife-107677-fig5-data4.zip › Figure 5—source data 4/Figure 5D-MgdE-EGFP-H3-1.tif]

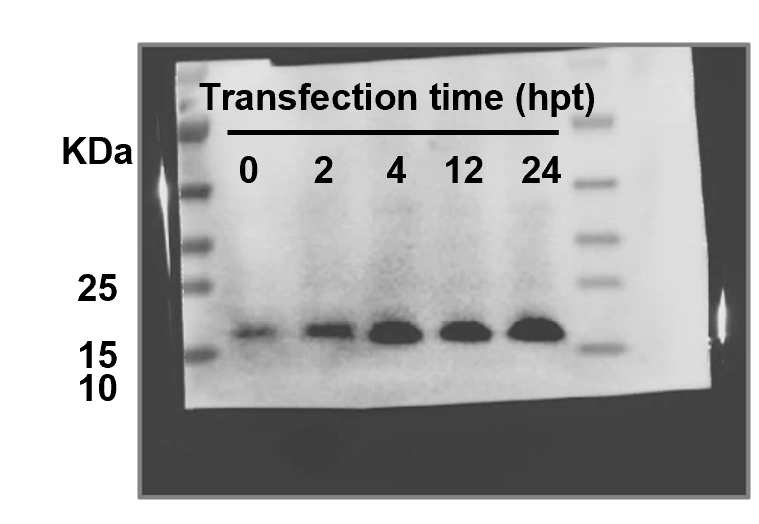

Supplement: Figure 5—source data 4. [file elife-107677-fig5-data4.zip › Figure 5—source data 4/Figure 5D-MgdE-EGFP-H3K4me3-1.tif]

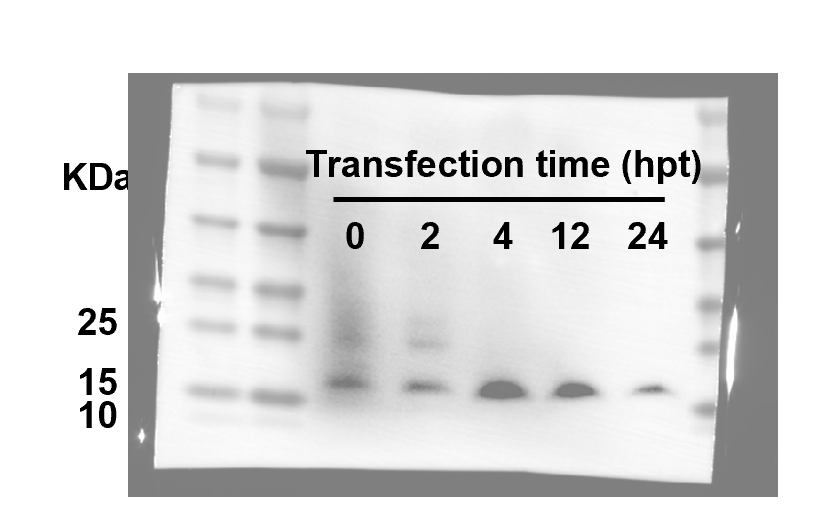

Supplement: Figure 5—source data 4. [file elife-107677-fig5-data4.zip › Figure 5—source data 4/Figure 5D-MgdE-EGFP-H3K4me3-2.tif]

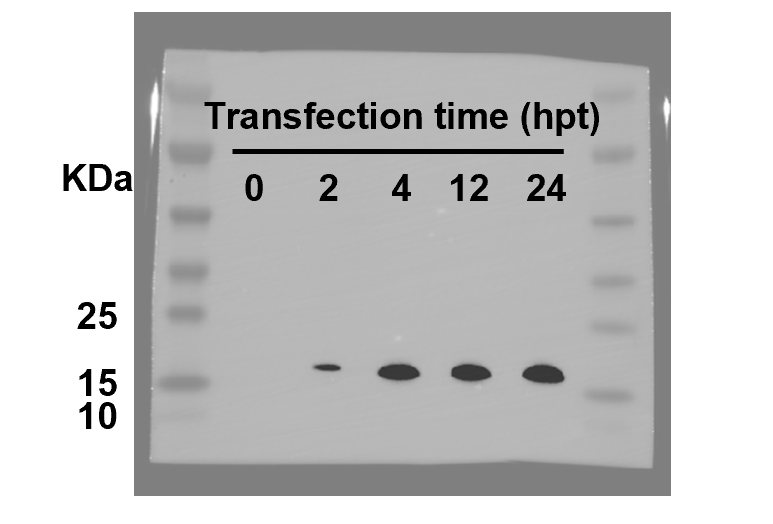

Supplement: Figure 5—source data 4. [file elife-107677-fig5-data4.zip › Figure 5—source data 4/Figure 5D-MgdE-EGFP-H3K4me3-3.tif]
